# Consumer Awareness, Perceptions and Acceptance of Mycoprotein Foods: An Exploratory Survey in China

**DOI:** 10.3390/foods15213783

**Published:** 2026-10-22

**Authors:** Zining Wang, Guobin Zhao, Kun Zhang, Yanzhe Yuan

**Affiliations:** 1 College of Economics and Management, Hefei Normal University, Hefei 230601, China; wangzining@hfnu.edu.cn; 2 Department of Tourism and Marketing, Griffith University, Southport, QLD 4215, Australia; guobin.zhao@griffithuni.edu.au (G.Z.); kun.zhang4@griffithuni.edu.au (K.Z.)

**Keywords:** mycoprotein, consumer awareness, consumer acceptance, food perceptions, cooking confidence, China

## Abstract

Understanding initial acceptance of mycoprotein foods requires examining consumers’ knowledge, product expectations and confidence in preparing them. An exploratory online survey of Chinese adults (*N* = 1197) assessed prior awareness and knowledge, followed by evaluations and acceptance intentions after a common description of mycoprotein production, food applications and ingredient-specific approval. Analyses comprised descriptive and conditional proportions, paired comparisons and multivariable regression. Most respondents had heard of mycoprotein (60.2%), but few reported high subjective knowledge (11.6%). After the description, willingness to try and purchase consideration were common (86.7% and 76.0%, respectively). Among willing-to-try respondents, low and moderate cooking confidence were reported by 22.2% and 29.1%. Overall impressions became more favourable across the survey sequence (*n* = 1069; paired mean difference = 0.334, 95% CI [0.296, 0.374]). Expected taste fit and curiosity were positively associated with both intentions. Nutritional expectations and cooking confidence were positively associated with purchase consideration, whereas worry was negatively associated. Safety, taste and nutrition were leading decision priorities. Initial acceptance encompassed different levels of knowledge and preparation confidence, identifying a need to consider consumers’ understanding and anticipated food use alongside acceptance correlates when planning product communication and testing.

## 1. Introduction

Alternative proteins are being developed to diversify food choices and provide options for reducing reliance on conventional meat. Their adoption, however, depends on consumers finding them understandable, appealing and suitable for everyday use. Research across protein sources identifies taste and health motives, familiarity, food neophobia, affective reactions and social context as recurring correlates of acceptance, with their relevance differing by product and population [1]. Studies of meat substitutes also show that recognising potential benefits does not necessarily overcome unfamiliarity or unfavourable sensory expectations [2,3]. For an emerging product category, understanding consumers’ starting points is therefore important before selecting marketing claims or testing purchase interventions.

Consumers’ understanding of the ingredient is relevant to their evaluations of mycoprotein. In this study, the term refers to the cultivated biomass of selected filamentous fungi, subsequently processed for food use. It is distinct from simply extracting protein from ordinary edible mushrooms, although consumers may associate these categories. Its protein and fibre content and filamentous structure provide a basis for developing foods with different nutritional and textural properties. Product studies have examined how mycoprotein formulations affect physicochemical characteristics and sensory acceptance: binding-agent formulations were optimised for nugget texture and sensory acceptance [4], whereas increasing mycoprotein incorporation in protein bars altered their fibre content, structure and sensory ratings [5]. These findings indicate that expectations about the ingredient need to be distinguished from sensory evaluations of a particular food formulation. Consumer research has also examined how people interpret fungi-based foods. Chezan et al. [6] documented familiarity, mould associations and sensory concerns in consumer responses to fungi-based protein, while Hellwig and Taherzadeh [7] examined public understanding of fungi-related terminology and production. These findings motivate examining what consumers associate with the name alongside their evaluations of foods made from an explicitly described mycoprotein ingredient.

China provides a timely setting for examining these initial consumer responses. In 2025, mycoprotein from *Fusarium venenatum* received approval as a novel food ingredient, subject to ingredient-specific conditions [8]. The introduction of this newly approved ingredient creates a practical need to establish what potential consumers already know, expect and want to learn about foods made from it. Previous research on alternative proteins highlights the relevance of product category and presentation to consumer acceptance. Bryant et al. [9] found differences in plant-based and cultured-meat acceptance across the United States, India and China, with familiarity positively associated with acceptance. Hartmann et al. [10] found differences between Chinese and German respondents in willingness to eat processed and unprocessed insect foods. Within China, Li et al. [11] showed that cultured-meat terminology and production information affected responses. A current assessment of mycoprotein can therefore complement earlier alternative-protein evidence by examining category understanding, anticipated food use and information needs in this changing regulatory context.

Research on mycoprotein acceptance has developed along two complementary lines: understanding how consumers interpret fungi-based foods and identifying the beliefs associated with their willingness to consume them. Food neophobia concerns reluctance to eat unfamiliar foods [12], while food technology neophobia concerns apprehension about foods produced using unfamiliar technologies [13]. In their fungi-based protein study, Chezan et al. [6] found that technology neophobia was related to perceived benefits and purchase intentions. Their neophobic and neophilic groups were defined using the mean technology-neophobia score, while interviews also revealed curiosity and excitement about trying fungal protein. These findings distinguish openness to unfamiliar food technologies from the immediate interest a specific food can evoke. Hellwig and Taherzadeh [7,14] examined appeal, concerns and understanding of fungi-related terminology and production. These studies show why familiarity with a name and understanding of a food category need to be considered separately. Research on product presentation also indicates that evaluations depend on the product concept being presented: Fischer and Hilboesen [15] examined substrate and processing information in mycelium products, and Lim and Tay [16] investigated the influence of serving-plate materials on mycoprotein perceptions. Interpreting these responses therefore requires attention to how consumers understand the food category and how the product is presented.

Other studies have examined which consumer beliefs are associated with mycoprotein acceptance. Dean et al. [17] used a multinational survey conducted in 2018/2019, including China, to examine willingness to try, buy and pay a premium for mycoprotein; healthiness, nutritional benefits, safety and sustainability were positively related to acceptance, whereas taste associations varied across their analyses. Lwin et al. [18] compared four alternative proteins in Singapore and identified familiarity, positive taste expectations and healthfulness beliefs as shared correlates of acceptance. These findings establish that both prior familiarity and anticipated product benefits warrant attention. However, knowledge-related associations do not imply that providing information will necessarily increase acceptance: Pawelczyk et al. [19] found no significant increase from health- or environment-focused messages alone, although knowledge, experience and technology neophobia were associated with mycoprotein acceptance. This distinction makes it important to characterise consumers’ existing understanding and information needs alongside their stated willingness.

Understanding the relationship between knowledge and acceptance requires distinguishing what consumers believe they know from the facts they recognise. Consumer research differentiates subjective knowledge, objective knowledge and usage experience because they can relate differently to decision making [20]. Piha et al. [21] applied this distinction to insect foods when examining knowledge, attitudes and willingness to buy. For mycoprotein, measuring self-assessed knowledge and recognition of specific production facts separately can clarify how each relates to initial acceptance. Whether consumers feel able to judge a product attribute is a further question: an overall willingness to try provides little detail about their expectations for taste. Examining explicit unable-to-judge responses alongside knowledge and intentions can therefore describe what receptive consumers know and which attributes they feel able to evaluate.

Anticipated food use adds a practical dimension to this assessment. Research on insect foods and meat substitutes links acceptance to curiosity, familiar meal combinations and preparation skills [22,23,24,25]. For mycoprotein foods, consumers may find the prospect of trying them appealing while differing in their confidence about preparing a meal with them. Trying and purchasing also deserve separate measurement: Chezan et al. [6] distinguished trying from regular-purchase intentions for fungal protein, as did Wilks and Phillips [26] for cultured meat. Finally, initial evaluations need to be interpreted in relation to the information supplied. Studies of cultured meat identify information valence, technical framing and naming as relevant to attitudes [11,27,28], alongside naturalness and disgust [29]. These findings support examining responses to a clearly described food concept, with attention to both anticipated qualities and preparation confidence.

Category-focused studies describe associations with fungal foods and understanding of their terminology and production [6,7,14], whereas acceptance models examine how product beliefs and familiarity relate to willingness [17,18]. A challenge in connecting these perspectives is that consumers may interpret the same category name differently, while a broad ingredient description leaves the sensory qualities and preparation of a specific product open. The present study examines what interested consumers know about mycoprotein and how they expect to use foods made from it. The specific question is whether receptive respondents share a similar understanding of mycoprotein, ability to evaluate its expected taste and confidence in preparing it. Describing these profiles can reveal differences within the receptive group that an overall acceptance estimate does not capture. Alongside these profiles, directly reported information requests identify topics consumers want to learn about, while the knowledge and preparation measures suggest directions for further product research. Examining acceptance correlates together with decision priorities additionally distinguishes attributes associated with willingness from what those respondents want considered in a decision.

Accordingly, this exploratory survey pursued three objectives: to describe Chinese respondents’ prior awareness, subjective knowledge, basic factual recognition and initial impressions of mycoprotein; to characterise evaluations and acceptance intentions following a common description of mycoprotein foods, including knowledge and preparation-confidence profiles among receptive respondents; and to examine associations of product expectations, cooking confidence, trial-related emotions and prior knowledge with willingness to try and purchase consideration. Decision priorities and information requests provide a complementary account of what respondents considered relevant to these choices. The study describes consumers’ interest in mycoprotein foods alongside what they understand about them, how they expect to use them and what they want to learn. Paired overall impressions provide supplementary evidence about how evaluations differed across the survey sequence.

## 2. Materials and Methods

### 2.1. Design, Recruitment and Participants

A single-session Chinese-language survey was administered through Credamo’s Data Marketplace (Credamo, Beijing, China) in September 2026. Eligibility required consent, age 18 years or above and usual residence in mainland China. Responses were excluded if consent was not provided or the age or residence criterion was not met. Of 1200 responses, one was excluded for lack of consent and two for not meeting the residence criterion; none were excluded on age grounds, leaving 1197 eligible respondents. Payment was CNY 2 per response, and recruitment used no demographic targeting or quotas.

The target was 1200 responses. For the descriptive aims, sample size can be evaluated in terms of estimation precision [30]: at this size, a proportion of 50% has an approximate 95% interval half-width of 2.8 percentage points under simple random sampling. This provides a precision benchmark for the overall proportions; the unweighted online sample is described in terms of its observed composition.

All eligible respondents completed the core closed-ended items. Checks identified no duplicate respondent identifiers or IP addresses and no conflicts in conditional routing or multiple-response selection limits. Respondents who answered the comprehension question incorrectly remained in the main analyses; descriptive results for those who answered correctly are provided as a sensitivity analysis (Section S3.3).

### 2.2. Questionnaire Sequence and Product Description

After screening, respondents reported whether they had previously heard the Chinese term for mycoprotein (*zhenjun danbai*), rated their subjective knowledge, and supplied a brief free association. Those who had heard of the term identified previous information channels. Respondents then rated their initial overall impression and answered two basic production-knowledge questions. All respondents then received the same description at the same point in the questionnaire. It defined the target ingredient as processed biomass obtained by cultivating selected filamentous fungi under controlled fermentation conditions. It described potential applications in patties, balls and fillings. The passage also stated that particular mycoprotein ingredients had received novel-food-ingredient approval in China, subject to ingredient-specific conditions and population restrictions.

The Supplementary Materials contain the complete English questionnaire, including this description (Section S2). Respondents evaluated a general food concept without a specified strain, brand, formulation or price. Following a comprehension question, respondents evaluated the described foods, their willingness to try and consider purchasing them, their information interest, anticipated attributes, cooking confidence and immediate emotions. Decision priorities, desired information, perceived previous consumption and demographic questions followed. All evaluations and intentions were questionnaire-based.

The approval statement is documented by Announcement No. 7 of 2025 for mycoprotein from *Fusarium venenatum* [8]. The annex specifies strains A3/5 or TB01 and ingredient-specific requirements, including population exclusions. Section S2.3 provides the exact description presented to respondents and separately identifies the additional regulatory details as researcher annotations.

### 2.3. Measures

The questionnaire used study-specific items informed by alternative-protein consumer research. Section S2.6 documents their purposes and conceptual sources. Most evaluations and intentions were assessed with single items; the two production questions formed a basic factual-recognition score. The questionnaire was not administered in a separate pilot or cognitive-interview study before launch, and no independent psychometric validation was conducted. In this paper, “inability to evaluate” denotes an explicit unable-to-evaluate or unable-to-judge response.

Prior awareness had three responses: heard of the term, unsure, and not heard of it. Subjective knowledge ranged from 1 (no knowledge) to 5 (very knowledgeable). The two production questions assessed recognition of cultivation and processing of fungal biomass, and recognition that fermentation can be used to produce mycoprotein ingredients. Correct answers were summed to a 0–2 factual recognition score.

Initial and post-description overall impressions used identical five-category response scales, from very negative (1) to very positive (5), with a separate option indicating that no impression had formed and an evaluation could not be made. The initial item referred to mycoprotein based on existing understanding; the later item referred to the mycoprotein foods in the description.

Willingness to try asked how willing respondents would be to try the foods once if they could conveniently obtain them. Purchase consideration asked how willing they would be to include the foods among the alternatives considered when purchasing. Both used five ordered categories from completely unwilling (1) to very willing (5), plus an unable-to-decide option. Scores 4–5 were classified as positive intentions for descriptive summaries and binary sensitivity analyses.

Expected taste fit, nutritional value, naturalness, food-safety risk and negative environmental impact were assessed with separate five-category items. Each offered an explicit unable-to-judge response. Cooking confidence asked how confident respondents were about combining or cooking the foods to prepare a meal. Curiosity, worry and aversion each used a single intensity item about trying the foods; aversion captured a broad negative reaction. Cooking confidence and the three emotional items had five ordered response categories without a separate uncertainty option. Full wording, response anchors and coding appear in Sections S2.3 and S2.4.

Two multiple-response questions asked respondents to select up to three decision concerns and up to three information topics they most wanted to learn about. These questions measured relative priorities within the supplied lists. Respondents also indicated whether, in light of the description, they believed they had previously eaten such foods. Those answering yes could identify the product, brand or place of consumption. These accounts were analysed descriptively as perceived prior consumption.

### 2.4. Statistical Analysis

Analyses were exploratory and comprised descriptive distributions, paired comparisons and multivariable associations. Figure S1 summarises sample eligibility, response coding and the analytical procedures. Unless otherwise stated, percentages used all 1197 eligible respondents as the denominator. Information-channel percentages used the 721 respondents who reported prior awareness. Means and standard deviations used only responses selecting an ordinal rating (hereafter, scorable responses); uncertainty was never coded as the scale midpoint. Multiselect percentages may sum to more than 100%.

Exploratory conditional proportions described knowledge and explicit inability to judge taste among respondents expressing positive responses, and acceptance within knowledge groups. Subjective knowledge was grouped by its verbal anchors: little or none (scores 1–2), some (score 3), and quite or very knowledgeable (scores 4–5). Factual-recognition profiles retained all three scores (0, 1 and 2). Additional profiles examined cooking confidence among respondents expressing positive intentions, perceived risk among those prioritising safety, and taste-information requests among those able or unable to judge taste. Cooking confidence was grouped as low (1–2), moderate (3) and high (4–5), with a combined moderate-or-lower category (1–3). Low perceived risk comprised scores 1–2. Taste was selected as the attribute with the most frequent unable-to-judge response. Proportions used pointwise Wilson 95% confidence intervals, which describe precision under the calculation’s assumptions rather than correcting for nonprobability selection. Full conditional results appear in Sections S3.7, S3.8 and S3.11.

The primary paired overall-impression comparison included the 1069 respondents who supplied a five-category evaluation at both assessments. An exact two-sided sign test compared increases with decreases, omitting ties from that test. The mean paired difference and its percentile bootstrap 95% confidence interval were estimated with 5000 respondent-level resamples. The standardised paired difference, *d*_z_, divided the mean difference by the standard deviation of individual differences. Mean differences and *d*_z_ treat scores as approximately equally spaced; the sign test uses only their ordering. The full transition table was also reported to preserve changes into and out of the unable-to-evaluate category. For full-sample positive-response comparisons, scores 4–5 were contrasted with all remaining responses, including inability to evaluate or decide. These paired binary proportions were compared using exact McNemar tests. Analogous paired procedures compared willingness to try with purchase consideration.

Separate ordinary least-squares (OLS) models examined the two intention outcomes, using HC3 heteroskedasticity-consistent standard errors [31]. The 11 substantive predictors covered four domains: anticipated product qualities (taste fit, nutrition, naturalness, safety risk and negative environmental impact), anticipated use (cooking confidence), trial-related emotions (curiosity, worry and aversion), and prior knowledge (subjective knowledge and factual recognition). Entering these domains together estimated their conditional associations with each intention. Pairwise Spearman correlations described the corresponding unadjusted associations, using scorable pairs and reporting their effective sample sizes (Section S3.10). Covariates were age, gender, student status, completed education, current residential area, dietary pattern and household income. Income bands were represented categorically. Education was grouped as below bachelor’s degree, bachelor’s degree, and master’s degree or above; diet was grouped as meat eating without active reduction, active meat reduction, and other patterns. Grouping limited sparse adjustment categories; the sample description retained all original categories.

For each of the five attribute items allowing an unable-to-judge response, the main model included a separate indicator for that category. A mean placeholder, calculated from available values in the eligible sample, was used in the numerical attribute column for unable-to-judge responses. This parameterisation estimates a linear score association among scorable responses and a separate level for unable-to-judge responses; the placeholder does not assign an attitude. Continuous predictor columns were centred and scaled in standard-deviation units; the five attribute items used the standard deviation of their scorable values in the corresponding analysis sample. Outcomes remained on the original 1–5 scale. Respondents unable to decide on an outcome were excluded only from models of that outcome, giving *n* = 1194 for trying and *n* = 1181 for purchase consideration. Binary covariates and categorical indicators were not standardised.

All non-intercept coefficient tests within each model were adjusted using the Benjamini–Hochberg (BH) procedure [32]. Tables report adjusted *p*-values alongside pointwise, unadjusted 95% confidence intervals. Normal asymptotic approximations were used for regression inference.

Sensitivity analyses used complete cases and logistic models comparing scores 4–5 with scores 1–3. Logistic models used sandwich standard errors and were fitted both to complete cases and to samples retaining unable-to-judge attribute responses through separate indicators. Descriptive proportions were also examined among respondents who answered the comprehension question correctly (Section S3.3). Diagnostics examined OLS leverage, Cook’s distance and exact leave-one-out coefficient changes, and logistic convergence, score gradients, information-matrix eigenvalues and fitted probabilities (Section S3.9). An additional complete-case model examined purchase-consideration scores minus trying scores using the same predictors to assess differences in associations across the two intentions.

**Figure 1:**
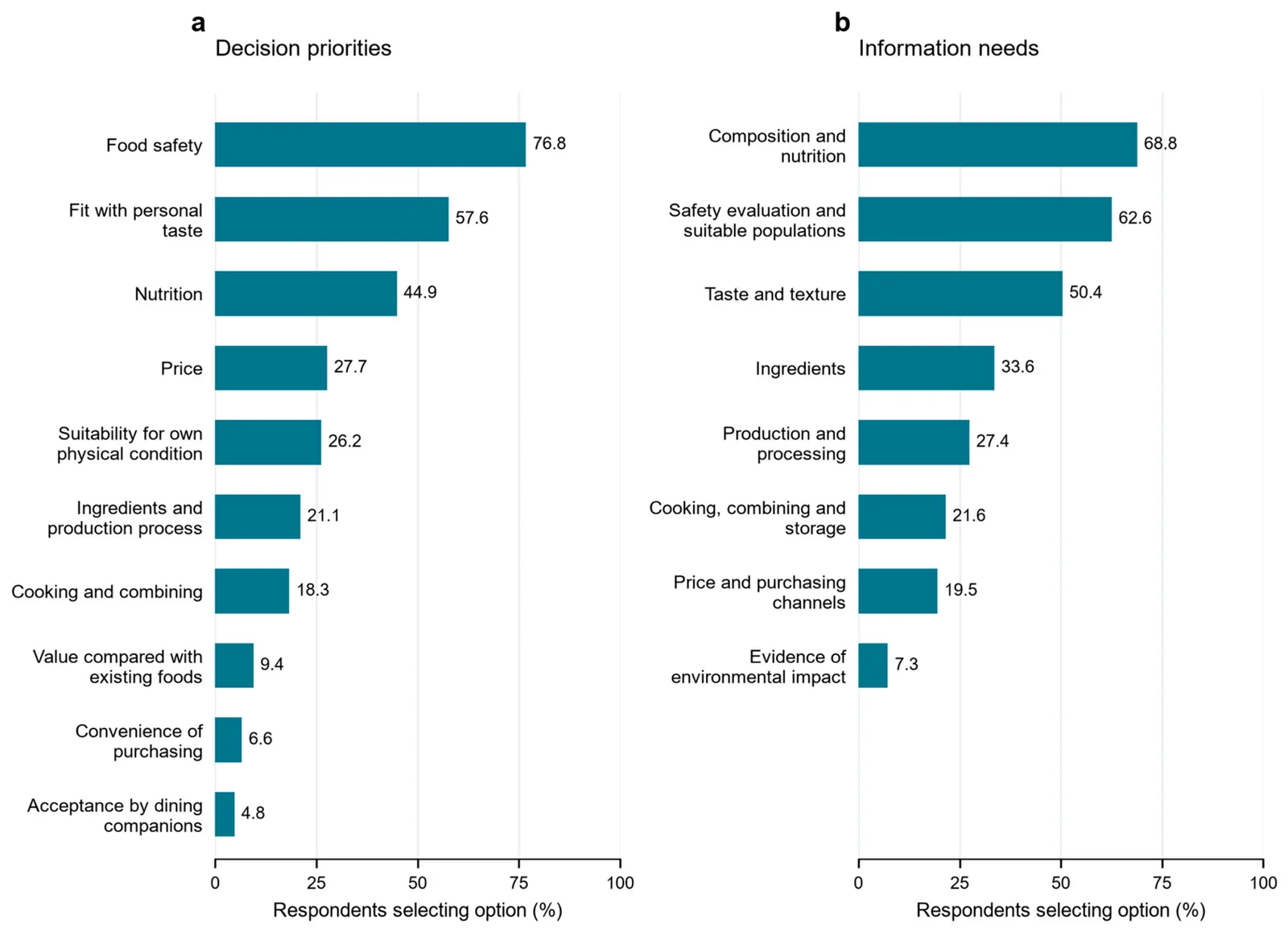
Decision priorities and information needs regarding mycoprotein foods. (**a**) Attributes prioritised when considering trying or purchasing the foods. (**b**) Information respondents most wanted to learn about. Bars show percentages of all respondents (*N* = 1197); up to three options could be selected per question. All substantive options are shown and ranked independently within each panel. Complete response distributions appear in Section S3.5.

**Figure 2:**
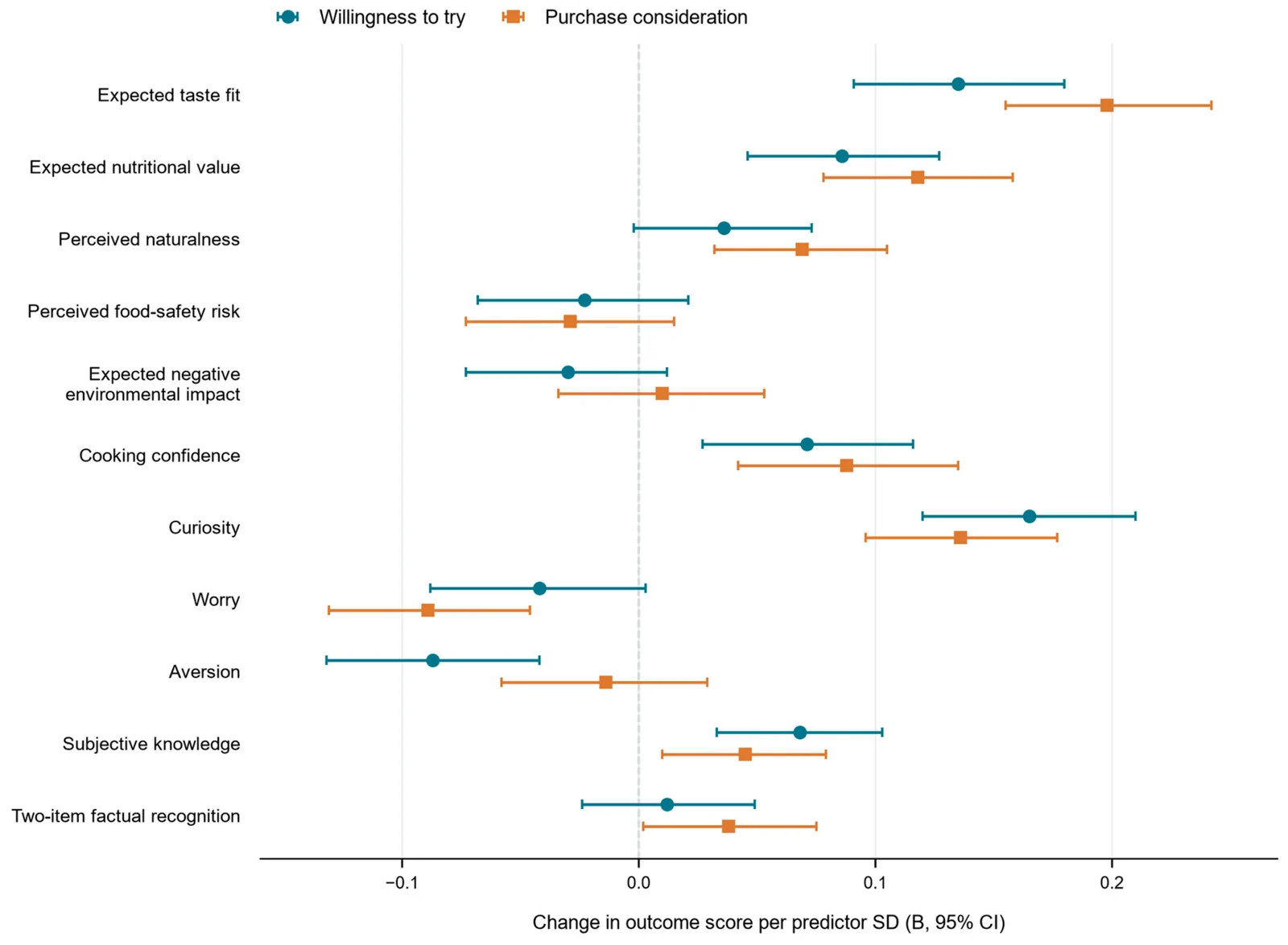
Multivariable associations with willingness to try and purchase consideration. Points show main-model OLS coefficients (trying: *n* = 1194; purchase consideration: *n* = 1181); horizontal lines show unadjusted 95% confidence intervals based on HC3 standard errors. *B* represents the change in outcome points per predictor *SD*. Section S3.4 reports all coefficients and Benjamini–Hochberg-adjusted *p*-values across the 30 non-intercept terms per model.

Free associations were screened using overlapping keyword rules defined during the initial descriptive analysis. Section S2.5 reports the rules, coverage and exact-match counts for brief no-association and knowledge-related answers. Analyses used Python 3.12.14, NumPy 2.3.5, pandas 3.0.1 and openpyxl 3.1.5. Figure 1 and Figure 2 were generated using Matplotlib version 3.11.2. Full sample distributions appear in Section S1 and complete statistical results in Section S3. Supplementary methods describe the coding rules (Sections S2.4 and S2.5) and paired-comparison computation (Section S3.6).

The original Chinese questionnaire, de-identified closed-ended response data, data dictionary, analysis code, aggregate results and figure source data are available through an OSF repository. The repository also provides the keyword-screening rules and aggregate findings for the open-ended responses.

## 3. Results

### 3.1. Sample Characteristics

Respondents were predominantly young adults, with a broad overall age range (*M* = 31.81 years, *SD* = 9.89; median = 29; range = 18–69). Women formed the majority (*n* = 733, 61.2%), as did respondents with at least a bachelor’s degree (*n* = 988, 82.5%) and those living in urban areas of cities or county seats (*n* = 1058, 88.4%); a smaller group were students (*n* = 201, 16.8%). Most ate meat without actively reducing it (*n* = 706, 59.0%) or were actively reducing meat consumption (*n* = 426, 35.6%). Supplementary Table S1.4 reports all observed demographic categories, including uncertain or withheld income. The two demographic options with no responses are identified in Section S1.3. Provincial-level counts appear in Supplementary Table S1.1.

### 3.2. Initial Awareness, Knowledge and Reported Experience

Most respondents had heard of mycoprotein (*n* = 721, 60.2%), but few reported high subjective knowledge (*n* = 139, 11.6%); little or no knowledge was more common (*n* = 726, 60.7%). Both production facts were recognised by 765 respondents (63.9%), while 177 selected extraction from edible mushrooms as the production method (14.8%). Complete distributions are reported in Section S1.3.

Among respondents with prior awareness, short videos or social media were the leading information channel (665/721, 92.2%), followed by news or popular-science information and commercial sources (Section S3.5).

The free-association screen most often matched nutrition or health (*n* = 137, 11.4%), edible mushrooms (*n* = 113, 9.4%), plants or soy (*n* = 106, 8.9%), and fermentation, mycelium or cultivation (*n* = 104, 8.7%). At least one keyword category matched 499 answers (41.7%); 164 answers indicated no association (13.7%). The remaining 534 answers (44.6%) matched neither the keyword categories nor the no-association expressions. Section S2.5 reports all categories, overlaps and unmatched answers.

After reading the description, some respondents reported perceived prior consumption of the target foods (*n* = 252, 21.1%), while more were unsure whether they had consumed them (n = 439, 36.7%). Keyword screening of their product accounts identified references to mushrooms, soy and yeast products (Section S2.5).

### 3.3. Changes in Overall Impressions Within the Survey

Overall-impression ratings were higher at the later assessment among respondents who supplied both ratings (*n* = 1069; before *M* = 3.70, *SD* = 0.70; after *M* = 4.04, *SD* = 0.63; paired mean difference = 0.334, bootstrap 95% CI [0.296, 0.374]; *d*_z_ = 0.509). Increases outnumbered decreases (380 versus 62, with 627 unchanged; exact sign test, *p* < 0.001). Positive ratings in this paired sample increased from 64.5% to 84.5% (237 moved into and 23 out of positive categories; exact McNemar test, *p* < 0.001).

Across all respondents, inability to express an overall impression fell from 125 to 8 (Table 1). Most initially unable to evaluate subsequently provided a rating (120/125), including 76 positive ratings. The full-sample positive proportion rose from 57.6% to 81.8% (difference = 24.2 percentage points, bootstrap 95% CI [21.6, 26.9]), combining changes in existing evaluations with newly expressed judgments.

### 3.4. Acceptance, Anticipated Attributes and Emotions

Positive willingness to try was more common than positive purchase consideration (1038/1197, 86.7%, versus 910/1197, 76.0%; exact McNemar test, *p* < 0.001; difference = 10.7 percentage points, bootstrap 95% CI [8.7, 12.8]). Among respondents who rated both intentions, purchase-consideration scores were lower (*n* = 1180; paired mean difference, purchase minus trying = −0.226, bootstrap 95% CI [−0.266, −0.187]). Table S1.5 reports the descriptive summaries.

Expectations were generally favourable: high nutritional value (79.8%), good taste fit (62.9%), low safety risk (70.7%) and low negative environmental impact (81.0%) were common. Fewer respondents reported high cooking confidence (43.4%). Strong curiosity was more frequent than strong worry or aversion (79.6%, 10.1% and 3.7%, respectively; Section S1.3 and Table S1.5).

Unable-to-judge responses were most frequent for expected taste fit (*n* = 147, 12.3%); unable-to-judge responses for the other attributes ranged from 1.7% to 5.6%.

### 3.5. Acceptance in Relation to Prior Knowledge and Preparation Confidence

Willingness to try increased across low, some and high subjective-knowledge groups (80.7%, 94.9% and 98.6%; Section S3.7). Nevertheless, respondents with little or no prior knowledge made up more than half of those willing to try (586/1038, 56.5%; Table 2).

Some respondents with favourable overall impressions could not judge expected taste fit (84/979, 8.6%; Table 2). Inability to judge taste was more frequent at lower subjective knowledge (16.5%, 7.5% and 1.4% across low, some and high knowledge groups; Section S3.7).

Most willing-to-try respondents recognised both production facts (701/1038, 67.5%). Both were also recognised by approximately half of those reporting little or no subjective knowledge (374/726, 51.5%), compared with 80.7% and 88.5% in the some- and high-knowledge groups (Section S3.8).

Low and moderate cooking confidence were both common among those willing to try (22.2% and 29.1%) and those considering purchase (16.5% and 29.8%; Table S3.11b). Moderate-or-lower confidence characterised about half of each group (51.3% and 46.3%; Table 2).

### 3.6. Decision Priorities and Information Needs

Most respondents expressed high interest in learning more about the foods (*n* = 1025, 85.6%; Table S1.5). Safety, taste and nutrition led respondents’ decision priorities (Figure 1). The most requested information concerned composition and nutrition, safety evaluation and suitable populations, and taste and texture; environmental-impact evidence was selected less often (*n* = 87, 7.3%). Low perceived risk was common even among those prioritising safety (619/919, 67.4%). Taste information was requested by similar proportions of respondents unable and able to judge taste fit (47.6% and 50.8%; Section S3.11).

### 3.7. Multivariable Associations and Sensitivity Analyses

The main OLS models retained unable-to-judge attribute responses (trying: *n* = 1194, *R*^2^ = 0.470; purchase consideration: *n* = 1181, *R*^2^ = 0.522). Figure 2 shows the substantive coefficients; Section S3.4 reports all estimates, and Sections S3.2 and S3.9 provide diagnostics.

Expected taste fit and curiosity were positively associated with both intentions. For trying, nutrition, cooking confidence and subjective knowledge also had positive associations, while aversion had a negative association. For purchase consideration, positive associations were observed for taste fit, nutrition, cooking confidence, curiosity, naturalness and subjective knowledge, while worry was negatively associated (Figure 2; Section S3.4).

Factual recognition, safety risk and negative environmental impact did not meet the BH-adjusted threshold in either model (Section S3.4). In the unadjusted analyses, factual recognition correlated positively with both intentions, whereas safety risk and negative environmental impact correlated negatively (Section S3.10).

Among respondents who rated purchase consideration, positive responses were more common when all five attribute ratings were available (818/990, 82.6%) than when at least one could not be judged (92/191, 48.2%).

Complete-case OLS estimates were similar to the main estimates. Logistic sensitivity analyses supported positive associations of taste fit and curiosity with both intentions, and of nutrition and cooking confidence with purchase consideration, together with a negative association for worry. For trying, the associations of nutritional expectations and cooking confidence did not meet the BH-adjusted threshold in either logistic model. Subjective knowledge for both intentions and naturalness for purchase consideration also did not meet this threshold. Aversion retained a negative trying association in the logistic model including unable-to-judge responses. No substantive predictor met the adjusted threshold in the purchase-minus-trying model (Section S3.4).

## 4. Discussion

### 4.1. Awareness and Understanding of Mycoprotein

Awareness of the name was common, but high subjective knowledge was uncommon, and factual recognition provided a different account of consumers’ understanding (Section S1.3). Approximately half of those reporting little or no knowledge nevertheless recognised both production facts (51.5%; Section S3.8). This combination is consistent with the distinction between subjective and objective knowledge in consumer research [20,21]: feeling unfamiliar with a food category does not necessarily mean failing to recognise basic facts about it. It also complements category-focused research on familiarity, mould associations and fungi-related terminology [6,7]. Consumer information can therefore address both the meaning of the ingredient and its practical applications, rather than treating all low-knowledge respondents as having the same informational starting point.

Free associations and perceived consumption accounts provide context for these differences in understanding. The keyword screen captured nutrition and health associations alongside familiar food categories and production-related terms. Keyword matches in accounts accompanying perceived prior consumption (21.1%) included mushrooms, soy and yeast products. These references suggest that familiar foods may serve as reference points for interpreting the ingredient. The food choice process model situates current choices within accumulated food experiences and personal resources [33]; from this perspective, associations with familiar foods offer a possible link to prior experience, which could be explored through interviews about specific products and food memories. The closed questions and open responses thus describe different aspects of consumers’ starting points: knowledge self-assessments, prompted recognition and spontaneous associations.

### 4.2. Interpreting the Change in Overall Impressions

The paired comparison documents two related changes in overall impressions (Table 1). Among respondents who rated overall impressions at both assessments, average scores became more positive and increases outnumbered decreases (*n* = 1069). Across the full sample, fewer selected the unable-to-evaluate option after the description (*N* = 1197). One finding concerns how ratings changed among respondents who provided both ratings; the other concerns whether respondents could provide a rating at all. The full transition table distinguishes changes in the direction of existing evaluations from the emergence of an expressed evaluation among initially uncertain respondents.

Prior research provides reasons to expect evaluations of unfamiliar foods to depend on information. Bekker et al. [27] found that information valence changed explicit cultured-meat attitudes using designs that addressed pretest sensitisation. Bryant and Dillard [28] found less favourable responses under a high-technology frame, and Li et al. [11] reported limited improvement following production information in China. Evidence on mycoprotein also indicates variation in responses to information: Pawelczyk et al. [19] did not find that health- or environment-focused messages alone significantly increased mycoprotein acceptance. These studies highlight the relevance of information content and presentation when interpreting evaluations of unfamiliar foods.

In the present survey, overall impressions were more favourable and more frequently expressed at the later assessment following the common description. The description combined a production explanation, food applications and an ingredient-specific regulatory statement; the later item also referred more explicitly to foods made from that ingredient. The observed difference reflects the survey sequence as a whole, including the description, conceptual clarification, intervening questions and repeated assessment. Without a randomised comparison, the contribution of information provision cannot be isolated.

### 4.3. Acceptance Intentions and Preparation Confidence

Positive willingness to try was more common than positive purchase consideration, including among respondents who provided ratings for both questions (Section 3.4; Section S3.6). Chezan et al. [6] reported higher trying than regular-purchase intentions for fungal protein, while Wilks and Phillips [26] distinguished trying cultured meat from consuming it regularly. Lwin et al. [18] combined everyday eating and regular purchasing in an acceptance index. Here, the comparison concerns responses to two specific questions: Q13 asked about trying once under convenient availability, whereas Q14 asked about inclusion in purchase consideration without repeating that availability condition. The observed difference may therefore reflect both commitment and wording conditions.

Initial receptiveness encompassed different levels of prior knowledge and preparation confidence (Table 2). Willingness to try was more prevalent at higher self-assessed knowledge levels, but was already common in the low-knowledge group (80.7%, 94.9% and 98.6% across the three levels). Little or no prior knowledge characterised more than half of those willing to try (56.5%; Table 2), indicating that limited self-assessed knowledge frequently coexisted with interest in the described foods. The profiles add to the acceptance associations reported by Dean et al. [17] and Lwin et al. [18] by describing the knowledge of respondents already willing to try the foods.

The explicit unable-to-judge responses further distinguish overall favourability from expectations about a specific attribute. Among respondents with favourable post-description impressions, a minority could not judge whether the foods would suit their taste (8.6%; Table 2). Positive category-level evaluations could thus coexist with an unresolved personal taste judgement. The broad ingredient description may have been sufficient for an overall impression while leaving the sensory properties of a particular formulation unclear. The category associations examined by Chezan et al. [6] and the terminology research of Hellwig and Taherzadeh [7] help explain why a broad food category can evoke different interpretations. Reporting unable-to-judge responses alongside taste ratings shows how often respondents could not assess this attribute.

Cooking confidence connects a favourable response to an anticipated meal-preparation task. Willingness to try concerns an initial encounter with the food, whereas cooking confidence concerns using it to prepare a meal. The trying question assumed convenient availability and did not require respondents to prepare the food themselves, so interest in trying could coexist with limited preparation confidence. Among those willing to try, 22.2% reported low cooking confidence and a further 29.1% reported moderate confidence (Table S3.11b). This distinction is consistent with the food choice process model, in which sensory and nutritional considerations are weighed alongside convenience and personal resources [33]. It also connects the present profiles to research on meal context [24], preparation-related constraints [25] and everyday use of unfamiliar foods [23].

### 4.4. Decision Priorities, Acceptance Correlates and Practical Implications

The decision priorities and information requests identify what respondents wanted to consider when evaluating these foods (Figure 1). Safety led respondents’ stated priorities, and many who prioritised it nevertheless expected little risk (619/919, 67.4%; Table S3.11a). A safety priority can therefore coexist with a relatively favourable risk assessment. The association models provide a complementary perspective (Figure 2): perceived risk and negative environmental impact correlated negatively with intentions, but their conditional associations were weak after adjustment (Table S3.10; Figure 2). Dean et al. [17] reported positive acceptance associations for safety and sustainability beliefs, whereas the association with green consumption values reported by Lwin et al. [18] concerned a broader personal orientation. Within the present survey, the priority questions identify matters respondents wanted considered in a decision, while the regression models describe how differences in attribute ratings related to differences in intentions. Safety therefore remained a communication priority despite its weak adjusted association with intentions.

The multivariable findings identify anticipated sensory appeal and curiosity as relevant to both trying and purchase consideration (Figure 2). The positive taste-fit association is consistent with mycoprotein evidence from Singapore [18] and with research on meat substitutes and insect foods [2,3,10], while Dean et al. [17] reported mixed taste associations. Nutritional expectations were also positively associated with both intention scores in the main models, complementing the nutritional and health-related findings of Dean et al. [17] and Lwin et al. [18]. Together with the cooking-confidence profiles, these associations suggest that initial interest concerns both how appealing the foods seem and how consumers expect to use them. The positive curiosity association points to an exploratory response to the prospect of trying the foods, consistent with the interest expressed in Chezan et al.’s interviews [6] and with the motivations for trying unfamiliar foods discussed by Tan et al. [22].

Worry and aversion describe the negative reactions accompanying this interest. Research on food and food technology neophobia situates avoidance of unfamiliar foods within broader individual dispositions [6,12,13], whereas the present items captured immediate responses to trying the described foods. In the main models, stronger aversion was associated with lower trying scores, while stronger worry was associated with lower purchase-consideration scores. The difference-score analysis did not establish a corresponding difference in these associations between intentions. Aversion represented a broad negative reaction, distinct from the disgust measures used in cultured-meat and mycelium research [15,29].

The findings suggest several directions for product development and communication testing (Figure 1 and Figure 2). Concrete descriptions of taste, texture and food applications can be paired with product trials to examine whether sensory experience matches expectations. Product-specific ingredient and nutritional information can address interest in composition and nutritional value, alongside the safety information respondents prioritised. Convenient formats, familiar meal applications and clear preparation steps, cooking times and recipe examples are relevant options for consumers interested in trying the foods but less confident about preparing them. Product demonstrations and small tasting portions offer ways to explore the role of curiosity in an initial encounter. Environmental-impact evidence attracted comparatively few selections under the three-choice limit; safety, nutrition and sensory information were more immediate informational priorities in this sample.

The high willingness to try provides a basis for recruiting consumers into trials with specific products. The convenient availability assumed by the trying item makes opportunities to encounter and obtain products relevant to such trials, while actual tasting can reveal whether experience meets expectations. Purchase consideration was assessed without a specified price; online purchase designs [34] and choice tasks incorporating prices and alternatives [35] can examine how initial interest relates to choices under these conditions. Lim and Tay [16] observed perception changes without a corresponding preference difference, and Hartmann and Siegrist [36] examined responses after tasting, illustrating the value of distinguishing presentation from product experience. Repeated product use can further examine whether evaluations change as consumers become familiar with a particular food.

### 4.5. Limitations

The online sample covered 31 provincial-level areas but was recruited without demographic quotas and included predominantly younger, highly educated and urban respondents. The relatively favourable acceptance levels should therefore be interpreted in the context of this sample composition; they may not be equally prevalent in older, less highly educated or rural groups that were less well represented. Geographic breadth does not establish national representativeness, and small numbers in some age, dietary and residential categories constrain subgroup interpretation.

The questionnaire assessed a broad food concept without specifying a brand, formulation, price or actual tasting experience. Intentions may differ across products and use occasions, and the description’s regulatory content may have influenced later responses. The cross-sectional associations remain open to reverse direction, shared response tendencies and unmeasured characteristics. The emotional items captured immediate responses; food and technology neophobia and broader social motives were unmeasured. These dispositions warrant attention given their role in previous research [6,18] and the availability of a food technology neophobia measure [13]. The environmental item assessed an anticipated product impact; broader green consumption values, distinguished from product beliefs by Lwin et al. [18] and characterised as a personal orientation by Haws et al. [37], were outside the measures used here.

Most attributes and intentions were measured with single items, limiting assessment of reliability and separation of related constructs. The two factual questions capture elementary prompted recognition. Explicit unable-to-judge responses indicate that a rating could not or would not be supplied under the available information; they do not capture uncertainty among respondents who chose a score. Limited experience, the broad product referent and reluctance to guess may each contribute. OLS treats score intervals as approximately equal and estimates linear attribute associations with separate levels for unable-to-judge responses. The complete-case and binary analyses assess sensitivity to the availability of ratings and the choice of outcome scale (Section S3).

Finally, keyword screening of open responses captures textual matches and provides limited insight into their intended meanings. Independent content coding, interviews, cognitive testing of category definitions and product verification would help clarify these associations and connect perceived familiarity with product experience.

## 5. Conclusions

This exploratory survey provides an account of how Chinese online respondents understood and initially evaluated mycoprotein foods. Awareness of the name was common, but high self-assessed knowledge was uncommon, and positive responses to the described foods encompassed different levels of knowledge and preparation confidence. Expected taste fit, nutritional value, cooking confidence and curiosity were positively associated with both intention scores in the main models; worry was negatively associated with purchase consideration. Safety, nutrition and sensory information remained prominent in respondents’ stated priorities. Beyond describing acceptance and its correlates, the study shows that receptive consumers differ in their knowledge and confidence in preparing the foods. These findings identify priorities for product communication and preparation support. Future sensory evaluations, trials with specific mycoprotein foods and studies of actual purchasing behaviour can examine how initial interest relates to product experience and subsequent food choices.

## Figures and Tables

**Table 1:** Transitions from initial to post-description overall impressions (counts).

Initial Impression	Very Negative	Somewhat Negative	Neither	Somewhat Positive	Very Positive	Unable to Evaluate	Total
Very negative	0	1	2	1	0	1	5
Somewhat negative	0	5	6	22	0	0	33
Neither	0	7	122	198	16	2	345
Somewhat positive	0	1	20	432	134	0	587
Very positive	0	0	2	32	68	0	102
Unable to evaluate	0	6	38	65	11	5	125
Total	0	20	190	750	229	8	1197

Note. Columns show post-description responses. “Neither” means neither positive nor negative.

**Table 2:** Selected knowledge, evaluation and preparation profiles among respondents.

Conditioning Group	Response Described	*n*/*N*	%	95% CI
Positive willingness to try	Little or no subjective knowledge	586/1038	56.5	[53.4, 59.4]
Little or no subjective knowledge	Both production facts recognised	374/726	51.5	[47.9, 55.1]
Positive post-description overall impression	Insufficient information to judge expected taste fit	84/979	8.6	[7.0, 10.5]
Positive willingness to try	Moderate or lower cooking confidence	532/1038	51.3	[48.2, 54.3]
Positive purchase consideration	Moderate or lower cooking confidence	421/910	46.3	[43.0, 49.5]

Note. Conditioning groups overlap. Confidence intervals are expressed in percentage units.

## Data Availability

The de-identified closed-ended survey data, data dictionary, original Chinese questionnaire, analysis code, aggregate results and figure source data supporting this study are available on the Open Science Framework (OSF) at https://osf.io/5f8yd/?view_only=2c76f40c41bb430cb24391bd48595ae9 (accessed on 28 September 2026). The repository also contains the keyword-screening rules and aggregate findings for the open-ended responses.

## Supplementary Materials

The following supporting information can be downloaded at: https://www.mdpi.com/article/10.3390/foods15213783/s1. Supplementary Material S1: Sample and response distributions; Supplementary Material S2: Full questionnaire and coding; Supplementary Material S3: Supplementary statistical results; Figure S1: Overview of sample eligibility, response coding and the statistical analyses; Table S1.1: Complete provincial-level distribution; Table S1.2: Respondent flow and data checks; Table S1.3: Complete categorical response distributions; Table S1.4: Complete sample characteristics; Table S1.5: Post-description evaluations and intentions; Table S3.7: Exploratory conditional descriptions of acceptance, knowledge and inability to judge taste; Table S3.8: Factual recognition by subjective knowledge and willingness to try; Table S3.10: Pairwise Spearman correlations with acceptance intentions; Table S3.11a: Conditional preparation, safety and information profiles; Table S3.11b: Cooking confidence among all respondents and those expressing positive intentions.
