# Supplementary material for: Consumer Awareness, Perceptions and Acceptance of Mycoprotein Foods: An Exploratory Survey in China
Source: Foods. 2026 Oct 22;15(21):3783. doi: 10.3390/foods15213783 (PMC13763357; doi:10.3390/foods15213783)
Supplement: Supplementary file 1 [file foods-15-03783-s001.zip › foods-4624109-supplementary.pdf]

Consumer awareness, perceptions and acceptance of mycoprotein foods: An exploratory survey in China

Contents

- S1: Sample composition and response distributions, including the complete demographic table (S1.4) and post-description summaries (S1.5).
- S2: Complete English reporting translation, routing, coding and item rationale.
- S3: Analytical flowchart (Figure S1), statistical specifications, supplementary results, knowledge profiles and diagnostics.
- S3 navigation: model definitions and fit (S3.1–S3.2); comprehension sensitivity (S3.3); main and sensitivity coefficients (S3.4); multiple-response distributions (S3.5); paired comparisons (S3.6); knowledge profiles (S3.7–S3.8); diagnostics (S3.9); correlations (S3.10); preparation and information profiles (S3.11).
- References.

Percentages and their confidence limits are reported to one decimal place; means and standard deviations to two; coefficients, standard errors, mean differences, effect sizes and R^2 to three; and VIF to two. Unless stated otherwise, percentages use all eligible respondents (N = 1,197). Reference numbers correspond to those in the main manuscript.

Supplementary Material S1: Sample and response distributions

Table S1.1. Complete provincial-level distribution

Self-reported usual residence (Q31), N = 1,197; no IP-based location assignment or weighting. Each respondent is counted once. Names identify administrative areas.

Provincial-level area	*n*	%
Guangdong	185	15.5
Shandong	90	7.5
Jiangsu	79	6.6
Henan	65	5.4
Hebei	56	4.7
Hubei	55	4.6
Zhejiang	53	4.4
Sichuan	51	4.3
Jiangxi	48	4.0
Hunan	44	3.7
Anhui	44	3.7
Shanxi	42	3.5
Shanghai	41	3.4

Provincial-level area	*n*	%
Guangxi	38	3.2
Heilongjiang	38	3.2
Liaoning	38	3.2
Shaanxi	29	2.4
Fujian	29	2.4
Beijing	25	2.1
Guizhou	24	2.0
Yunnan	23	1.9
Jilin	19	1.6
Tianjin	18	1.5
Chongqing	17	1.4
Gansu	16	1.3
Inner Mongolia	11	0.9
Xinjiang	10	0.8
Hainan	4	0.3
Ningxia	3	0.3
Qinghai	1	0.1
Xizang (Tibet)	1	0.1

Table S1.2. Respondent flow and data checks

Check	Result
Exported records	1,200
Did not agree to participate	1
Did not meet mainland-residence criterion	2
Eligible sample	1,197
Age range	18–69 years
Median completion time	359 s
Completion-time interquartile range	266–456 s
Comprehension question not correct	10
Duplicate respondent identifiers among eligible records	0
Duplicate IP addresses among eligible records	0
Core closed-ended item nonresponse	0

Explicit uncertainty selections were retained as substantive responses. The checks cover exported records; invitation and non-completion totals were unavailable for calculation of a response rate.

Table S1.3. Complete categorical response distributions

Percentages use all eligible respondents ($N = 1,197$). All response options are shown, including zero counts. Complete demographic distributions are in Table S1.4 and provincial-level counts in Table S1.1. No respondent selected "Do not know" for residential area or "Prefer not to answer" for education (both $n = 0$; 0.0%). Multiple-response distributions are in S3.5. Age is summarised continuously in the manuscript and grouped in Table S1.4.

Q4. Before taking this survey, had you heard the term "mycoprotein"?

Response	*n*	%
Never heard of it	231	19.3
Not sure whether I have heard of it	245	20.5
Have heard of it	721	60.2

Q5. Before this survey, how much did you think you knew about mycoprotein?

Response	*n*	%
No knowledge at all	215	18.0
Very little knowledge	511	42.7
Some knowledge	332	27.7
Quite knowledgeable	122	10.2
Very knowledgeable	17	1.4

Q8. Based only on your current understanding, what is your overall impression of mycoprotein?

Response	*n*	%
Very negative	5	0.4
Somewhat negative	33	2.8
Neither positive nor negative	345	28.8
Somewhat positive	587	49.0
Very positive	102	8.5
I have not yet formed an impression and cannot evaluate it	125	10.4

Q9. How is what is commonly called mycoprotein in the food sector mainly obtained? Answer from existing knowledge without looking up information.

Response	*n*	%
Extracting protein from ordinary edible mushrooms	177	14.8
Cultivating selected fungi and processing their biomass	877	73.3
Extracting protein from soybeans	46	3.8
Cultivating animal cells	6	0.5
Do not know	91	7.6

Q10. Fermentation cultivation can be used to produce mycoprotein food ingredients. Is this statement correct?

**Response**	***n***	**%**
True	923	77.1
False	25	2.1
Do not know	249	20.8

Q11. How is the mycoprotein ingredient just described mainly obtained?

**Response**	***n***	**%**
Cultivating fungi and processing their biomass	1,187	99.2
Cultivating animal cells	3	0.3
Processing soybeans	4	0.3
I did not understand clearly	3	0.3

Q12. Based on the description above, what is your overall impression of these mycoprotein foods?

**Response**	***n***	**%**
Very negative	0	0.0
Somewhat negative	20	1.7
Neither positive nor negative	190	15.9
Somewhat positive	750	62.7
Very positive	229	19.1
I have not yet formed an impression and cannot evaluate it	8	0.7

Q13. Assuming that you could conveniently obtain these mycoprotein foods, how willing are you currently to try them once?

**Response**	***n***	**%**
Completely unwilling	4	0.3
Somewhat unwilling	31	2.6
No clear inclination either way	121	10.1
Somewhat willing	676	56.5
Very willing	362	30.2
Currently unable to decide	3	0.3

Q14. How willing are you currently to include these mycoprotein foods among the alternatives you consider when purchasing?

**Response**	***n***	**%**
Completely unwilling	9	0.8
Somewhat unwilling	33	2.8
No clear inclination either way	229	19.1
Somewhat willing	677	56.6
Very willing	233	19.5
Currently unable to decide	16	1.3

Q15. How much would you currently like to learn more about these mycoprotein foods?

**Response**	***n***	**%**
Not at all	2	0.2
Not very much	9	0.8
Moderately	161	13.5
Quite a lot	705	58.9
Very much	320	26.7

Q16. To what extent do you expect the taste of these mycoprotein foods to suit your taste preferences?

**Response**	***n***	**%**
Not at all a good fit	4	0.3
Not a very good fit	36	3.0
Moderate fit	257	21.5
Quite a good fit	632	52.8
Very good fit	121	10.1
Insufficient information to judge	147	12.3

Q17. What nutritional value do you expect these mycoprotein foods to have?

**Response**	***n***	**%**
Very low	5	0.4
Rather low	21	1.8
Moderate	185	15.5
Rather high	663	55.4
Very high	292	24.4
Insufficient information to judge	31	2.6

Q18. How natural do you think these mycoprotein foods are?

**Response**	***n***	**%**
Not at all natural	25	2.1
Not very natural	171	14.3
Moderately natural	317	26.5
Quite natural	490	40.9
Very natural	174	14.5
Insufficient information to judge	20	1.7

Q19. How large do you think the food-safety risk of eating these mycoprotein foods is?

**Response**	***n***	**%**
Very small	106	8.9
Rather small	740	61.8
Moderate	231	19.3
Rather large	61	5.1
Very large	12	1.0
Insufficient information to judge	47	3.9

Q20. How large a negative environmental impact do you expect the production of these mycoprotein foods to have?

**Response**	***n***	**%**
Very small	349	29.2
Rather small	621	51.9
Moderate	108	9.0
Rather large	44	3.7
Very large	8	0.7
Insufficient information to judge	67	5.6

Q21. If you used these mycoprotein foods to prepare a meal, how confident would you be about how to cook them or combine them with other foods?

**Response**	***n***	**%**
Not at all confident	54	4.5
Not very confident	289	24.1
Moderately confident	335	28.0
Quite confident	486	40.6
Very confident	33	2.8

Q22. When thinking about trying these mycoprotein foods, how much curiosity do you currently feel?

**Response**	***n***	**%**
None at all	4	0.3
Weak	56	4.7
Moderate	184	15.4
Strong	737	61.6
Very strong	216	18.0

Q23. When thinking about trying these mycoprotein foods, how much worry do you currently feel?

**Response**	***n***	**%**
None at all	90	7.5
Weak	729	60.9
Moderate	257	21.5
Strong	103	8.6
Very strong	18	1.5

Q24. When thinking about trying these mycoprotein foods, how much aversion do you currently feel?

**Response**	***n***	**%**
None at all	503	42.0
Weak	548	45.8
Moderate	102	8.5
Strong	40	3.3
Very strong	4	0.3

Q27. Based on the description, do you think you have previously actually eaten the mycoprotein foods meant here?

**Response**	***n***	**%**
No	506	42.3
Not sure	439	36.7
Yes	252	21.1

Table S1.4. Complete sample characteristics

**Characteristic**	**Category**	***n***	**%**
Age group (years)	18–29	606	50.6
Age group (years)	30–44	441	36.8
Age group (years)	45–59	135	11.3
Age group (years)	60–69	15	1.3
Gender	Female	733	61.2
Gender	Male	464	38.8
Highest completed education	Bachelor's degree	824	68.8
Highest completed education	Master's degree or above	164	13.7
Highest completed education	Junior college	130	10.9
Highest completed education	Senior secondary or vocational secondary	62	5.2
Highest completed education	Junior secondary or below	17	1.4
Highest completed education	Prefer not to answer	0	0.0
Current residential area	Urban area of a city or county seat	1,058	88.4
Current residential area	Town area outside the urban district	90	7.5
Current residential area	Rural area	49	4.1
Current residential area	Do not know	0	0.0
Currently a student	No	996	83.2
Currently a student	Yes	201	16.8
Dietary pattern	Eats meat; not actively reducing	706	59.0
Dietary pattern	Eats meat; actively reducing	426	35.6
Dietary pattern	No meat or aquatic foods; consumes eggs/dairy	24	2.0

**Characteristic**	**Category**	***n***	**%**
Dietary pattern	No meat; consumes fish/other aquatic foods	21	1.8
Dietary pattern	Other dietary pattern	16	1.3
Dietary pattern	No animal-derived foods	4	0.3
Monthly household disposable income (CNY)	6,000–9,999	305	25.5
Monthly household disposable income (CNY)	10,000–14,999	251	21.0
Monthly household disposable income (CNY)	3,000–5,999	209	17.5
Monthly household disposable income (CNY)	15,000–19,999	177	14.8
Monthly household disposable income (CNY)	≥20,000	153	12.8
Monthly household disposable income (CNY)	<3,000	86	7.2
Monthly household disposable income (CNY)	Prefer not to answer	9	0.8
Monthly household disposable income (CNY)	Do not know	7	0.6

Note. Percentages are unweighted. Residential area is current residence; education is completed attainment; income is household disposable income. Age groups were created for reporting.

Table S1.5. Post-description evaluations and intentions.

Item	Scorable *n*	*M* (*SD*)	Scores 4–5, *n* (%)	Unable to judge/decide, *n* (%)
Post-description overall impression	1,189	4.00 (0.65)	979 (81.8)	8 (0.7)
Willingness to try	1,194	4.14 (0.72)	1,038 (86.7)	3 (0.3)
Purchase consideration	1,181	3.92 (0.75)	910 (76.0)	16 (1.3)
Interest in further information	1,197	4.11 (0.66)	1,025 (85.6)	Not offered
Expected taste fit	1,050	3.79 (0.70)	753 (62.9)	147 (12.3)
Expected nutritional value	1,166	4.04 (0.72)	955 (79.8)	31 (2.6)
Perceived naturalness	1,177	3.52 (0.98)	664 (55.5)	20 (1.7)
Perceived food-safety risk	1,150	2.25 (0.73)	73 (6.1)	47 (3.9)
Expected negative environmental impact	1,130	1.89 (0.78)	52 (4.3)	67 (5.6)
Cooking confidence	1,197	3.13 (0.96)	519 (43.4)	Not offered
Curiosity	1,197	3.92 (0.74)	953 (79.6)	Not offered
Worry	1,197	2.36 (0.80)	121 (10.1)	Not offered
Aversion	1,197	1.74 (0.78)	44 (3.7)	Not offered

Note. Percentages use all 1,197 respondents. Higher scores indicate more of each named attribute. "Not offered" means that the item had no unable-to-judge/decide option.

Supplementary Material S2: Full questionnaire and coding

S2.1. Source and reading guide

The questionnaire was administered in Chinese. This English translation, prepared after data collection, retains all items, response options, selection limits and routing rules. Numbering follows Q1–Q35; the product description appears between Q10 and Q11. Section headings and researcher notes were added for reporting.

Closed-ended questions allowed one response unless otherwise stated. Options retain their original order. Eligibility and conditional-display rules are given with the relevant questions; the wording and required-item discrepancy for Q28 is noted there.

S2.2. Participant introduction

Consumer awareness and attitudes towards mycoprotein: A survey

Welcome to this survey about views on mycoprotein foods. This study examines people's understanding of mycoprotein food ingredients and products made from them; it does not involve actual consumption or purchasing. You are welcome to participate whether or not you are familiar with these foods. Please answer according to your current understanding, without looking up information online. The information collected will be used only for academic research. Participation is entirely voluntary, and you may stop answering at any time. Please do not provide personal information, such as your real name or telephone number, in open-ended answers.

S2.3. Questions and information block

Q1. Do you agree to participate in this research?

- I agree

- I do not agree

Response format/routing: Routing: selecting "I do not agree" ends the questionnaire.

Q2. What is your age in completed years?

Numeric entry: age in completed years.

Response format/routing: Routing: an age below 18 ends the questionnaire.

Q3. Do you currently usually reside in mainland China?

- Yes
- No

Response format/routing: Routing: selecting "No" ends the questionnaire.

Q4. Before taking this survey, had you heard the term "mycoprotein"?

- Never heard of it
- Not sure whether I have heard of it
- Have heard of it

Q5. Before this survey, how much did you think you knew about mycoprotein?

- No knowledge at all
- Very little knowledge
- Some knowledge
- Quite knowledgeable
- Very knowledgeable

Q6. What first comes to mind when you see the term "mycoprotein"? Give a brief thought; write none if you have no association.

Open-ended text entry.

Q7. Through which channels had you previously encountered information about mycoprotein?

- Short videos or social media
- News reports or popular-science information
- E-commerce, packaging or advertising
- Physical shops or restaurants
- School courses, academic articles or work
- Family or friends
- Other
- Cannot remember

Response format/routing: Display only if Q4 = "Have heard of it". Multiple responses allowed (up to eight options). "Cannot remember" is exclusive and cannot be combined with other options.

Q8. Based only on your current understanding, what is your overall impression of mycoprotein?

- Very negative
- Somewhat negative
- Neither positive nor negative
- Somewhat positive

- Very positive
- I have not yet formed an impression and cannot evaluate it

Q9. How is what is commonly called mycoprotein in the food sector mainly obtained? Answer from existing knowledge without looking up information.

- Extracting protein from ordinary edible mushrooms
- Cultivating selected fungi and processing their biomass
- Extracting protein from soybeans
- Cultivating animal cells
- Do not know

Q10. Fermentation cultivation can be used to produce mycoprotein food ingredients. Is this statement correct?

- True
- False
- Do not know

Common description of mycoprotein production, food applications and ingredient-specific approval

The mycoprotein discussed in this questionnaire is a food ingredient made by cultivating selected filamentous fungi under controlled fermentation conditions and then processing the resulting fungal biomass. This ingredient can be combined with other ingredients to make foods such as patties, balls and fillings. In recent years, certain mycoprotein ingredients have been approved for use as novel food ingredients in China. Specific ingredients are subject to corresponding conditions of use and provisions concerning suitable consumer groups. In the following questions, please evaluate the general concept of these mycoprotein foods.

Researcher note. The administered stimulus contained the regulatory statement shown above without citing a specific document. For source documentation, see National Health Commission Announcement No. 7 of 2025, which approved mycoprotein from *Fusarium venenatum* [8]. Its annex identifies A3/5 or TB01 as production strains and excludes infants and young children, pregnant or lactating women, and people allergic to edible fungi. This annotation was added for reporting; these details were not separately shown to participants.

Q11. How is the mycoprotein ingredient just described mainly obtained?

- Cultivating fungi and processing their biomass
- Cultivating animal cells
- Processing soybeans
- I did not understand clearly

Q12. Based on the description above, what is your overall impression of these mycoprotein foods?

- Very negative
- Somewhat negative
- Neither positive nor negative
- Somewhat positive
- Very positive
- I have not yet formed an impression and cannot evaluate it

Q13. Assuming that you could conveniently obtain these mycoprotein foods, how willing are you currently to try them once?

- Completely unwilling
- Somewhat unwilling
- No clear inclination either way
- Somewhat willing
- Very willing
- Currently unable to decide

Q14. How willing are you currently to include these mycoprotein foods among the alternatives you consider when purchasing?

- Completely unwilling
- Somewhat unwilling
- No clear inclination either way
- Somewhat willing
- Very willing
- Currently unable to decide

Q15. How much would you currently like to learn more about these mycoprotein foods?

- Not at all
- Not very much
- Moderately
- Quite a lot
- Very much

Q16. To what extent do you expect the taste of these mycoprotein foods to suit your taste preferences?

- Not at all a good fit
- Not a very good fit
- Moderate fit
- Quite a good fit
- Very good fit
- Insufficient information to judge

Q17. What nutritional value do you expect these mycoprotein foods to have?

- Very low
- Rather low
- Moderate
- Rather high
- Very high
- Insufficient information to judge

Q18. How natural do you think these mycoprotein foods are?

- Not at all natural
- Not very natural
- Moderately natural
- Quite natural

- Very natural
- Insufficient information to judge

Q19. How large do you think the food-safety risk of eating these mycoprotein foods is?

- Very small
- Rather small
- Moderate
- Rather large
- Very large
- Insufficient information to judge

Q20. How large a negative environmental impact do you expect the production of these mycoprotein foods to have?

- Very small
- Rather small
- Moderate
- Rather large
- Very large
- Insufficient information to judge

Q21. If you used these mycoprotein foods to prepare a meal, how confident would you be about how to cook them or combine them with other foods?

- Not at all confident
- Not very confident
- Moderately confident
- Quite confident
- Very confident

Q22. When thinking about trying these mycoprotein foods, how much curiosity do you currently feel?

- None at all
- Weak
- Moderate
- Strong
- Very strong

Q23. When thinking about trying these mycoprotein foods, how much worry do you currently feel?

- None at all
- Weak
- Moderate
- Strong
- Very strong

Q24. When thinking about trying these mycoprotein foods, how much aversion do you currently feel?

- None at all
- Weak
- Moderate

- Strong
- Very strong

Q25. If considering trying or purchasing these mycoprotein foods, which aspects currently matter most to you? Select up to three.

- Fit with my taste preferences
- Food safety
- Suitability for my own physical condition
- Nutrition
- Ingredients and production process
- Price
- Convenience of purchasing
- How to cook it or combine it with other foods
- Acceptance by people I eat with
- Value compared with existing foods
- Other
- Nothing in particular matters to me at present
- I cannot say at present

Response format/routing: Select up to three options. Each of the last two options is exclusive and cannot be combined with any other option.

Q26. Which information about these mycoprotein foods would you most like to learn more about? Select up to three.

- What the ingredients are
- Production and processing methods
- Composition and nutrition
- Safety evaluation and suitable consumer groups
- Taste and texture
- Cooking, combining with other foods and storage
- Price and purchasing channels
- Evidence of environmental impact
- Other
- I do not currently want further information

Response format/routing: Select up to three options. The last option is exclusive and cannot be combined with other options.

Q27. Based on the description, do you think you have previously actually eaten the mycoprotein foods meant here?

- No
- Not sure
- Yes

Q28. What product did you eat? You may give its name, brand or where you ate it. Leave blank if you cannot remember; do not provide personal information.

Open-ended text entry.

Response format/routing: Display only if Q27 = "Yes". The instruction permits leaving the answer blank, but the PDF displays a required-item marker; the export does not resolve whether blank submission was permitted.

Q29. Which option best describes your current dietary pattern?

- I eat meat or poultry and am not actively reducing my meat consumption
- I eat meat or poultry but am actively reducing my meat consumption
- I do not eat meat or poultry but eat fish or other aquatic foods
- I do not eat meat, poultry or aquatic foods but eat eggs or dairy products
- I do not eat foods of animal origin
- Other dietary pattern

Q30. What is your gender?

- Male
- Female

Q31. In which provincial-level area do you currently usually reside?

- Beijing
- Tianjin
- Hebei
- Shanxi
- Inner Mongolia
- Liaoning
- Jilin
- Heilongjiang
- Shanghai
- Jiangsu
- Zhejiang
- Anhui
- Fujian
- Jiangxi
- Shandong
- Henan
- Hubei
- Hunan
- Guangdong
- Guangxi
- Hainan
- Chongqing
- Sichuan
- Guizhou
- Yunnan
- Xizang (Tibet)
- Shaanxi
- Gansu
- Qinghai

- Ningxia
- Xinjiang

Q32. Which type of area do you currently live in? Answer for current residence, not household registration.

- Urban area of a city or county seat
- Town area outside the urban district
- Rural area
- Do not know

Q33. What is your highest completed level of education?

- Junior secondary school or below
- Senior secondary school or vocational secondary education
- Junior college
- Bachelor’s degree
- Master’s degree or above
- Prefer not to answer

Q34. Are you currently a student?

- Yes
- No

Q35. Approximately what is your average monthly household disposable income? This means household income available for consumption and saving; an exact calculation is not necessary.

- Below CNY 3,000
- CNY 3,000–5,999
- CNY 6,000–9,999
- CNY 10,000–14,999
- CNY 15,000–19,999
- CNY 20,000 or more
- Do not know
- Prefer not to answer

S2.4. Scoring and interpretation

For Q5, Q8 and Q12–Q24, the five substantive response categories were scored from 1 to 5 in their listed order. The sixth categories in Q8, Q12–Q14 and Q16–Q20 were retained as separate uncertainty responses, not scored as zero or as the midpoint. Q15, Q21 and Q22–Q24 offered no uncertainty option. Scores of 4–5 indicate higher levels of the named response; they do not indicate a favourable evaluation for risk, environmental harm, worry or aversion.

Q9 was scored correct for “Cultivating selected fungi and processing their biomass”; Q10 was scored correct for “True”. Each correct answer contributed one point to the factual recognition score (0–2); all other responses, including “Do not know”, contributed zero. Q11 was correct for “Cultivating fungi and processing their biomass”. It was a comprehension check after the description, not part of the factual recognition score measured beforehand. Comprehension failures remained in the main sample.

Q8 asked about mycoprotein based on existing understanding, whereas Q12 asked about foods made from the ingredient just described. Their identical response anchors permit a paired comparison of overall

impressions, but the product referent and information available were not identical. The comparison cannot isolate an information effect.

Q13 measures willingness to try once under convenient availability, and Q14 measures inclusion in purchase consideration. Q24 records aversion as a broad negative reaction. The attribute items concern anticipated food qualities, and Q27 records perceived prior consumption.

S2.5. Exploratory screening of open responses

All 1,197 eligible respondents supplied a nonblank answer to Q6. The initial descriptive analysis used eight analyst-defined keyword rules covering food categories, production and commonly discussed attributes. These exploratory rules were developed after data collection. They match literal Chinese substrings after trimming surrounding whitespace. Each response counts once per matching category and may enter several categories. The table provides English glosses of the keyword rules. Coverage is reported against all 1,197 responses.

Screen	English glosses of search terms	Matched responses	%
Edible mushrooms	mushroom; edible fungi; shiitake; wood ear; enoki; edible fungus	113	9.4
Plants or soy	plant; soybean; soy product	106	8.9
Fermentation or cultivation	fermentation; mycelium; cultivation	104	8.7
Nutrition or health	nutrition; health; high protein	137	11.4
Bacteria, viruses or infection	bacteria; virus; infection	24	2.0
Yeast	yeast	8	0.7
Environment or sustainability	environmental protection; sustainable; environment	35	2.9
Meat alternatives	artificial meat; alternative meat; vegetarian meat; plant-based meat	54	4.5

At least one category matched 499 responses (41.7%); 68 responses matched more than one (5.7%). The eight rules left 698 responses unmatched (58.3%). A separate exact-match screen found 164 answers meaning “none”, “no” or “none at present” (13.7%; respectively 163, 1 and 0). Exact answers meaning “do not know”, “do not understand” or “unclear” occurred zero times in this screen. These two sets are reported

separately because they have different meanings. The remaining 534 responses (44.6%) were outside the keyword and exact-match sets. Counts describe matches to this rule set; the rules do not classify negation or the full meaning of an answer.

Q28 screening covered the 252 respondents reporting prior consumption in Q27. Screening trimmed surrounding whitespace and counted literal substring matches, allowing an account to match multiple sets. The sets cover Quorn and three Chinese name variants; the term mycoprotein; mushroom names (mushroom, shiitake, enoki, wood ear, matsutake, king oyster mushroom, bolete, wild mushroom and white mushroom); and a combined soy-or-yeast set (yeast, tofu, soy products, soybean and natto). Aggregate matches provide context for perceived consumption; the manuscript describes references to mushrooms, soy and yeast products at this level of classification.

S2.6. Item purpose and measurement rationale

The questionnaire was developed for this exploratory survey using concepts from alternative-protein consumer studies. The table identifies the purpose and literature context of each item group; the wording was not transferred verbatim from validated scales. Most ratings are single-item measures, and the factual-recognition score combines two questions about production. No separate pilot study, cognitive interviews or independent psychometric validation preceded the survey. The post-description comprehension question checks recognition of the supplied explanation. The complete administered wording is translated in S2.3.

Items	Purpose of the wording	Relevant conceptual context
Q4–Q6, Q9–Q10, Q27–Q28	Separate recognition of the category name, perceived knowledge, spontaneous associations, prompted factual recognition and perceived consumption experience.	Raju et al. [20]; Chezan et al. [6]; Hellwig and Taherzadeh [7].
Q8, Q12	Record the direction of overall impressions before and after the common description, while retaining an explicit unformed-impression response.	The comparison addresses the survey's descriptive research question.
Q13–Q14	Distinguish willingness to try once under convenient availability from willingness to include the foods in purchase consideration.	Chezan et al. [6]; Wilks and Phillips [26].
Q15–Q20	Assess information interest and anticipated taste fit, nutrition, naturalness, safety risk and environmental harm; attribute items permit inability to judge.	Hoek et al. [2]; Michel et al. [3]; Lwin et al. [18].
Q21	Assess confidence about preparing the described foods.	Elzerman et al. [24]; Schösler et al. [25].
Q22–Q24	Record immediate curiosity, worry and aversion specifically about trying.	Tan et al. [22].
Q25–Q26	Identify relative decision and information priorities within supplied lists using a maximum of three selections.	Study-specific descriptive questions.
Q29–Q35	Describe dietary and sociodemographic composition and provide observed covariates.	Descriptive categories and observed covariates.

Supplementary Material S3: Supplementary statistical results

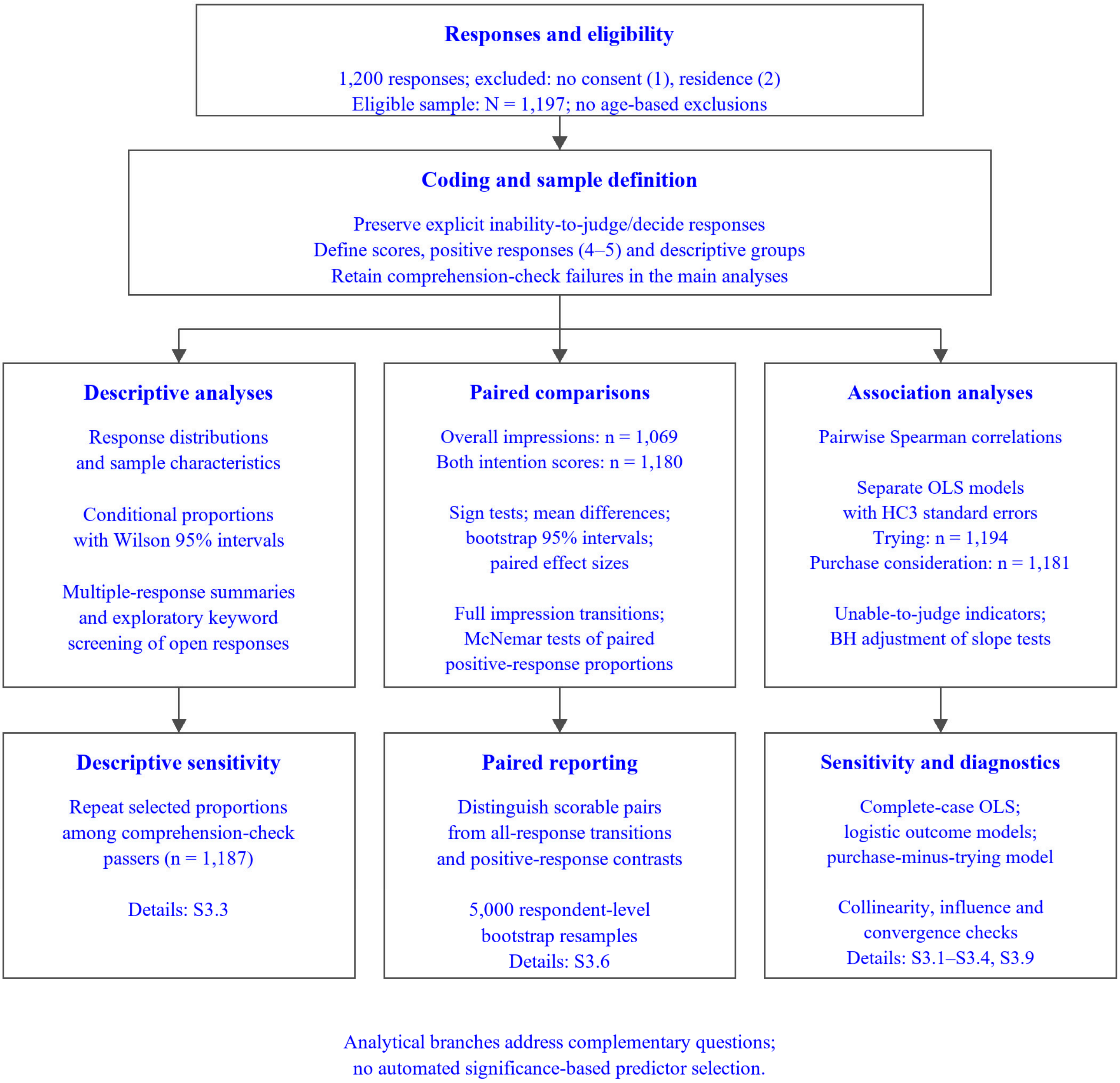

Figure S1. Overview of sample eligibility, response coding and the statistical analyses. OLS, ordinary least squares; HC3, heteroskedasticity-consistent covariance estimator; BH, Benjamini–Hochberg adjustment.

S3.1. Regression specifications

The two unable-to-judge-inclusive ordinary least-squares (OLS) models are the main association analyses. Complete-case OLS and binary logistic models assess sensitivity to sample composition and outcome coding. The additional purchase-minus-trying model examines differences in associations between intentions. S3.10 provides pairwise correlations; S3.11 provides preparation and information-priority profiles.

The main models contain 30 slopes plus an intercept: 11 substantive continuous predictors, age, gender, student status, two dietary indicators, two education indicators, urban residence, five income-band

indicators, one unknown/withheld-income indicator, and five unable-to-judge attribute indicators. Complete-case OLS and logistic models omit the final five unable-to-judge indicators. The income reference category is below CNY 3,000 per month. Coefficients for continuous substantive predictors and age are per *SD*; indicator coefficients are category differences. The dependent variable remains on its 1–5 scale in OLS. Main-model sample sizes are 1,194 (trying) and 1,181 (purchase consideration).

Unable-to-judge-inclusive models use a numerical attribute column with a mean placeholder and a separate indicator for inability to judge. The indicator estimates a separate level for that category. Respondents unable to decide on an outcome are excluded from models for that outcome.

OLS covariance is HC3. In the logistic models, the outcome is 1 for scores 4–5 and 0 for scores 1–3; unable-to-decide outcomes are excluded. Logistic covariance uses the sandwich estimator multiplied by n/(n − k), where k is the number of parameters including the intercept. Regression tests and intervals use normal approximations. Benjamini–Hochberg (BH) correction includes all slopes within each fitted model. Raw *p*-values and BH-adjusted *p*-values are supplied; displayed intervals are not multiplicity-adjusted. Logistic odds ratios (ORs) and their intervals are exponentiated coefficients and coefficient intervals. Predictors were not selected by automated significance screening.

S3.2. Model fit and collinearity

**Model**	**Outcome**	***N***	**Parameters including intercept**	***R*²**	**Maximum single-column VIF**
Complete-case OLS (HC3)	Trying	994	26	.425	4.00
Complete-case logistic	Trying	994	26	—	4.00
Unable-to-judge-inclusive OLS (main)	Trying	1,194	31	.470	3.94
Unable-to-judge-inclusive logistic	Trying	1,194	31	—	3.94
Complete-case OLS (HC3)	Purchase consideration	990	26	.485	4.04
Complete-case logistic	Purchase consideration	990	26	—	4.04
Unable-to-judge-inclusive OLS (main)	Purchase consideration	1,181	31	.522	3.94
Unable-to-judge-inclusive logistic	Purchase consideration	1,181	31	—	3.94
Purchase-minus-trying difference-score OLS	Purchase minus trying	990	26	.050	4.04

R^2 values are in-sample. The VIF values describe collinearity in the fitted design matrices. S3.9 reports case-influence and logistic-convergence diagnostics; model assumptions and outcome specifications are described in the manuscript.

S3.3. Sensitivity of descriptive proportions

**Sample**	***N***	**Positive trying (%)**	**Positive purchase consideration (%)**	**Positive post-description impression (%)**
All eligible	1,197	86.7	76.0	81.8
Comprehension passed	1,187	86.9	76.2	82.1

S3.4. Complete non-intercept coefficients

All non-intercept coefficients are reported below, with main OLS models followed by sensitivity analyses and the difference-score model. For logistic models, effects and intervals are on the odds-ratio scale, whereas SE is on the log-odds scale. Tables provide raw and BH-adjusted *p*-values; confidence intervals are pointwise.

Unable-to-judge-inclusive OLS (main) — Trying (*n* = 1,194)

**Predictor**	***B* [95% CI]**	***SE* (coefficient scale)**	**Raw *p***	**BH-adjusted *p***
Expected taste fit	0.135 [0.091, 0.180]	0.023	< .001	< .001
Expected nutritional value	0.086 [0.046, 0.127]	0.021	< .001	< .001
Perceived naturalness	0.036 [−0.002, 0.073]	0.019	.061	.231
Perceived food-safety risk	−0.023 [−0.068, 0.021]	0.023	.309	.707
Negative environmental impact	−0.030 [−0.073, 0.012]	0.022	.161	.438
Cooking confidence	0.071 [0.027, 0.116]	0.023	.002	.008
Curiosity	0.165 [0.120, 0.210]	0.023	< .001	< .001
Worry	−0.042 [−0.088, 0.003]	0.023	.070	.233
Aversion	−0.087 [−0.132, −0.042]	0.023	< .001	< .001
Subjective knowledge	0.068 [0.033, 0.103]	0.018	< .001	< .001
Factual recognition	0.012 [−0.024, 0.049]	0.019	.513	.734
Age	−0.003 [−0.040, 0.034]	0.019	.879	.977
Female (reference: male)	0.029 [−0.037, 0.095]	0.034	.394	.707
Student	0.069 [−0.031, 0.168]	0.051	.177	.443
Meat reduction (reference: not reducing)	−0.017 [−0.084, 0.051]	0.035	.630	.756

Predictor	*B* [95% CI]	*SE* (coefficient scale)	Raw *p*	BH-adjusted *p*
Other diet (reference: meat without reduction)	−0.070 [−0.226, 0.086]	0.080	.377	.707
Bachelor’s (reference: below bachelor’s)	−0.021 [−0.105, 0.063]	0.043	.623	.756
Master’s or above (reference: below bachelor’s)	0.046 [−0.066, 0.157]	0.057	.424	.707
Urban (reference: town/rural)	−0.034 [−0.137, 0.069]	0.053	.514	.734
Income 3,000–5,999 (reference: <3,000)	0.000 [−0.136, 0.136]	0.070	1.000	1.000
Income 6,000–9,999 (reference: <3,000)	0.056 [−0.080, 0.191]	0.069	.420	.707
Income 10,000–14,999 (reference: <3,000)	0.004 [−0.132, 0.140]	0.069	.956	.999
Income 15,000–19,999 (reference: <3,000)	0.064 [−0.078, 0.206]	0.072	.378	.707
Income ≥20,000 (reference: <3,000)	0.047 [−0.103, 0.197]	0.076	.538	.734
Income unknown/withheld (reference: <3,000)	0.008 [−0.344, 0.359]	0.179	.966	.999
Expected taste fit: unable to judge	−0.027 [−0.133, 0.078]	0.054	.610	.756
Expected nutritional value: unable to judge	−0.048 [−0.258, 0.163]	0.107	.657	.759
Perceived naturalness: unable to judge	−0.090 [−0.361, 0.181]	0.138	.516	.734
Perceived food-safety risk: unable to judge	−0.174 [−0.355, 0.007]	0.093	.060	.231
Negative environmental impact: unable to judge	−0.128 [−0.294, 0.039]	0.085	.132	.397

Unable-to-judge-inclusive OLS (main) — Purchase consideration (*n* = 1,181)

Predictor	*B* [95% CI]	*SE* (coefficient scale)	Raw *p*	BH-adjusted *p*
Expected taste fit	0.198 [0.155, 0.242]	0.022	< .001	< .001

Predictor	*B* [95% CI]	*SE* (coefficient scale)	Raw *p*	BH-adjusted *p*
Expected nutritional value	0.118 [0.078, 0.158]	0.020	< .001	< .001
Perceived naturalness	0.069 [0.032, 0.105]	0.019	< .001	.001
Perceived food-safety risk	−0.029 [−0.073, 0.015]	0.022	.203	.412
Negative environmental impact	0.010 [−0.034, 0.053]	0.022	.668	.802
Cooking confidence	0.088 [0.042, 0.135]	0.024	< .001	.001
Curiosity	0.136 [0.096, 0.177]	0.021	< .001	< .001
Worry	−0.089 [−0.131, −0.046]	0.022	< .001	< .001
Aversion	−0.014 [−0.058, 0.029]	0.022	.519	.676
Subjective knowledge	0.045 [0.010, 0.079]	0.018	.011	.047
Factual recognition	0.038 [0.002, 0.075]	0.019	.040	.134
Age	0.014 [−0.022, 0.049]	0.018	.447	.610
Female (reference: male)	0.000 [−0.066, 0.066]	0.034	.997	.997
Student	−0.058 [−0.151, 0.036]	0.048	.226	.412
Meat reduction (reference: not reducing)	0.060 [−0.007, 0.127]	0.034	.078	.195
Other diet (reference: meat without reduction)	−0.023 [−0.168, 0.122]	0.074	.754	.837
Bachelor’s (reference: below bachelor’s)	0.015 [−0.072, 0.102]	0.044	.734	.837
Master’s or above (reference: below bachelor’s)	−0.014 [−0.129, 0.100]	0.058	.809	.837
Urban (reference: town/rural)	−0.096 [−0.191, 0.000]	0.049	.050	.141
Income 3,000–5,999 (reference: <3,000)	−0.065 [−0.211, 0.081]	0.075	.384	.576
Income 6,000–9,999 (reference: <3,000)	−0.094 [−0.240, 0.051]	0.074	.203	.412
Income 10,000–14,999 (reference: <3,000)	−0.159 [−0.306, −0.012]	0.075	.033	.125
Income 15,000–19,999 (reference: <3,000)	−0.081 [−0.237, 0.075]	0.080	.311	.490

Predictor	*B* [95% CI]	***SE* (coefficient scale)**	Raw *p*	BH-adjusted *p*
Income ≥20,000 (reference: <3,000)	−0.096 [−0.259, 0.067]	0.083	.250	.416
Income unknown/withheld (reference: <3,000)	−0.179 [−0.472, 0.115]	0.150	.234	.412
Expected taste fit: unable to judge	−0.047 [−0.160, 0.067]	0.058	.421	.601
Expected nutritional value: unable to judge	−0.163 [−0.420, 0.095]	0.131	.216	.412
Perceived naturalness: unable to judge	−0.036 [−0.308, 0.235]	0.138	.793	.837
Perceived food-safety risk: unable to judge	−0.045 [−0.226, 0.136]	0.092	.627	.783
Negative environmental impact: unable to judge	−0.157 [−0.315, 0.001]	0.081	.052	.141

Complete-case OLS (HC3) — Trying ($n = 994$)

Predictor	*B* [95% CI]	***SE* (coefficient scale)**	Raw *p*	BH-adjusted *p*
Expected taste fit	0.125 [0.079, 0.170]	0.023	< .001	< .001
Expected nutritional value	0.088 [0.046, 0.130]	0.022	< .001	< .001
Perceived naturalness	0.038 [−0.001, 0.078]	0.020	.059	.211
Perceived food-safety risk	−0.013 [−0.060, 0.034]	0.024	.587	.803
Negative environmental impact	−0.034 [−0.079, 0.011]	0.023	.142	.394
Cooking confidence	0.072 [0.024, 0.120]	0.025	.003	.014
Curiosity	0.159 [0.112, 0.207]	0.024	< .001	< .001
Worry	−0.025 [−0.074, 0.025]	0.025	.326	.627
Aversion	−0.079 [−0.128, −0.031]	0.025	.001	.007
Subjective knowledge	0.074 [0.036, 0.112]	0.019	< .001	< .001
Factual recognition	0.010 [−0.030, 0.051]	0.021	.621	.803
Age	0.010 [−0.031, 0.051]	0.021	.632	.803
Female (reference: male)	−0.003 [−0.075, 0.069]	0.037	.932	.932

**Predictor**	***B* [95% CI]**	***SE* (coefficient scale)**	**Raw *p***	**BH-adjusted *p***
Student	0.104 [−0.010, 0.218]	0.058	.073	.229
Meat reduction (reference: not reducing)	−0.005 [−0.079, 0.069]	0.038	.892	.929
Other diet (reference: meat without reduction)	−0.061 [−0.232, 0.110]	0.087	.485	.757
Bachelor’s (reference: below bachelor’s)	−0.062 [−0.156, 0.032]	0.048	.195	.486
Master’s or above (reference: below bachelor’s)	0.023 [−0.097, 0.143]	0.061	.706	.803
Urban (reference: town/rural)	−0.063 [−0.175, 0.050]	0.057	.273	.569
Income 3,000–5,999 (reference: <3,000)	−0.015 [−0.171, 0.140]	0.079	.846	.920
Income 6,000–9,999 (reference: <3,000)	0.087 [−0.068, 0.241]	0.079	.272	.569
Income 10,000–14,999 (reference: <3,000)	0.033 [−0.123, 0.190]	0.080	.677	.803
Income 15,000–19,999 (reference: <3,000)	0.067 [−0.095, 0.229]	0.082	.416	.693
Income ≥20,000 (reference: <3,000)	0.075 [−0.095, 0.245]	0.087	.386	.689
Income unknown/withheld (reference: <3,000)	−0.083 [−0.509, 0.343]	0.217	.703	.803

Complete-case logistic — Trying (n = 994)

**Predictor**	**OR [95% CI]**	***SE* (coefficient scale)**	**Raw *p***	**BH-adjusted *p***
Expected taste fit	2.184 [1.479, 3.225]	0.199	< .001	.001
Expected nutritional value	1.433 [0.984, 2.086]	0.192	.061	.280
Perceived naturalness	1.215 [0.868, 1.701]	0.172	.257	.642
Perceived food-safety risk	0.979 [0.667, 1.438]	0.196	.915	.987
Negative environmental impact	0.786 [0.540, 1.144]	0.192	.208	.642
Cooking confidence	1.475 [0.987, 2.206]	0.205	.058	.280

**Predictor**	**OR [95% CI]**	***SE* (coefficient scale)**	**Raw *p***	**BH-adjusted *p***
Curiosity	2.457 [1.801, 3.351]	0.158	< .001	< .001
Worry	0.994 [0.670, 1.475]	0.201	.976	.987
Aversion	0.679 [0.449, 1.028]	0.212	.067	.280
Subjective knowledge	1.211 [0.779, 1.883]	0.225	.395	.779
Factual recognition	1.528 [1.128, 2.070]	0.155	.006	.052
Age	0.813 [0.549, 1.203]	0.200	.300	.682
Female (reference: male)	1.524 [0.741, 3.133]	0.368	.252	.642
Student	0.759 [0.305, 1.891]	0.465	.554	.793
Meat reduction (reference: not reducing)	1.389 [0.608, 3.173]	0.422	.436	.779
Other diet (reference: meat without reduction)	1.009 [0.338, 3.013]	0.558	.987	.987
Bachelor's (reference: below bachelor's)	0.746 [0.308, 1.806]	0.451	.516	.793
Master's or above (reference: below bachelor's)	1.412 [0.429, 4.649]	0.608	.571	.793
Urban (reference: town/rural)	0.669 [0.253, 1.771]	0.497	.418	.779
Income 3,000–5,999 (reference: <3,000)	0.791 [0.198, 3.164]	0.708	.740	.925
Income 6,000–9,999 (reference: <3,000)	1.670 [0.418, 6.675]	0.707	.468	.780
Income 10,000–14,999 (reference: <3,000)	0.967 [0.211, 4.438]	0.777	.966	.987
Income 15,000–19,999 (reference: <3,000)	2.925 [0.504, 16.961]	0.897	.231	.642
Income ≥20,000 (reference: <3,000)	1.189 [0.242, 5.836]	0.812	.831	.987
Income unknown/withheld (reference: <3,000)	1.674 [0.148, 18.905]	1.237	.677	.891

Unable-to-judge-inclusive logistic — Trying (n = 1,194)

**Predictor**	**OR [95% CI]**	***SE* (coefficient scale)**	**Raw *p***	**BH-adjusted *p***
Expected taste fit	2.155 [1.510, 3.077]	0.182	< .001	< .001
Expected nutritional value	1.614 [1.135, 2.295]	0.180	.008	.058
Perceived naturalness	1.132 [0.845, 1.515]	0.149	.407	.610
Perceived food-safety risk	0.864 [0.623, 1.199]	0.167	.383	.604
Negative environmental impact	0.805 [0.582, 1.114]	0.165	.191	.382
Cooking confidence	1.391 [1.020, 1.898]	0.159	.037	.145
Curiosity	2.322 [1.759, 3.064]	0.142	< .001	< .001
Worry	0.816 [0.606, 1.100]	0.152	.182	.382
Aversion	0.642 [0.474, 0.869]	0.155	.004	.042
Subjective knowledge	1.317 [0.942, 1.841]	0.171	.107	.267
Factual recognition	1.318 [1.037, 1.676]	0.122	.024	.120
Age	0.759 [0.564, 1.020]	0.151	.068	.184
Female (reference: male)	2.078 [1.174, 3.677]	0.291	.012	.072
Student	0.677 [0.324, 1.414]	0.376	.299	.526
Meat reduction (reference: not reducing)	1.060 [0.587, 1.912]	0.301	.848	.908
Other diet (reference: meat without reduction)	0.825 [0.316, 2.152]	0.489	.694	.833
Bachelor’s (reference: below bachelor’s)	0.969 [0.511, 1.839]	0.327	.924	.940
Master’s or above (reference: below bachelor’s)	1.936 [0.750, 5.002]	0.484	.172	.382
Urban (reference: town/rural)	0.922 [0.414, 2.053]	0.408	.842	.908
Income 3,000–5,999 (reference: <3,000)	0.744 [0.235, 2.352]	0.587	.615	.802
Income 6,000–9,999 (reference: <3,000)	1.189 [0.380, 3.719]	0.582	.766	.884
Income 10,000–14,999 (reference: <3,000)	0.675 [0.206, 2.210]	0.605	.516	.737

Predictor	OR [95% CI]	*SE* (coefficient scale)	Raw *p*	BH-adjusted *p*
Income 15,000–19,999 (reference: <3,000)	2.261 [0.523, 9.776]	0.747	.275	.515
Income ≥20,000 (reference: <3,000)	0.951 [0.255, 3.549]	0.672	.940	.940
Income unknown/withheld (reference: <3,000)	1.823 [0.226, 14.731]	1.066	.573	.782
Expected taste fit: unable to judge	0.527 [0.266, 1.043]	0.348	.066	.184
Expected nutritional value: unable to judge	0.346 [0.127, 0.946]	0.513	.039	.145
Perceived naturalness: unable to judge	0.736 [0.193, 2.814]	0.684	.655	.818
Perceived food-safety risk: unable to judge	0.461 [0.201, 1.054]	0.422	.066	.184
Negative environmental impact: unable to judge	0.681 [0.321, 1.443]	0.383	.316	.526

Complete-case OLS (HC3) — Purchase consideration ($n = 990$)

Predictor	*B* [95% CI]	*SE* (coefficient scale)	Raw *p*	BH-adjusted *p*
Expected taste fit	0.192 [0.148, 0.237]	0.023	< .001	< .001
Expected nutritional value	0.119 [0.077, 0.160]	0.021	< .001	< .001
Perceived naturalness	0.070 [0.030, 0.109]	0.020	< .001	.003
Perceived food-safety risk	−0.016 [−0.063, 0.031]	0.024	.512	.675
Negative environmental impact	0.007 [−0.040, 0.054]	0.024	.780	.848
Cooking confidence	0.085 [0.036, 0.135]	0.025	< .001	.003
Curiosity	0.115 [0.074, 0.157]	0.021	< .001	< .001
Worry	−0.092 [−0.136, −0.047]	0.023	< .001	< .001
Aversion	−0.012 [−0.059, 0.036]	0.024	.631	.752
Subjective knowledge	0.051 [0.013, 0.089]	0.019	.008	.029
Factual recognition	0.030 [−0.010, 0.070]	0.020	.136	.262

**Predictor**	***B* [95% CI]**	***SE* (coefficient scale)**	**Raw *p***	**BH-adjusted *p***
Age	0.018 [−0.021, 0.057]	0.020	.372	.547
Female (reference: male)	−0.001 [−0.072, 0.070]	0.036	.976	.976
Student	−0.034 [−0.138, 0.070]	0.053	.521	.675
Meat reduction (reference: not reducing)	0.068 [−0.005, 0.140]	0.037	.067	.168
Other diet (reference: meat without reduction)	−0.032 [−0.189, 0.125]	0.080	.690	.784
Bachelor's (reference: below bachelor's)	0.007 [−0.089, 0.103]	0.049	.886	.923
Master's or above (reference: below bachelor's)	−0.039 [−0.162, 0.085]	0.063	.540	.675
Urban (reference: town/rural)	−0.117 [−0.220, −0.014]	0.053	.027	.083
Income 3,000–5,999 (reference: <3,000)	−0.112 [−0.276, 0.051]	0.083	.178	.317
Income 6,000–9,999 (reference: <3,000)	−0.125 [−0.287, 0.037]	0.083	.130	.262
Income 10,000–14,999 (reference: <3,000)	−0.180 [−0.344, −0.015]	0.084	.032	.090
Income 15,000–19,999 (reference: <3,000)	−0.087 [−0.261, 0.086]	0.088	.323	.505
Income ≥20,000 (reference: <3,000)	−0.110 [−0.291, 0.071]	0.092	.235	.392
Income unknown/withheld (reference: <3,000)	−0.293 [−0.635, 0.050]	0.175	.094	.213

Complete-case logistic — Purchase consideration (*n* = 990)

**Predictor**	**OR [95% CI]**	***SE* (coefficient scale)**	**Raw *p***	**BH-adjusted *p***
Expected taste fit	2.071 [1.546, 2.775]	0.149	< .001	< .001
Expected nutritional value	1.737 [1.296, 2.329]	0.150	< .001	.002
Perceived naturalness	1.304 [1.025, 1.658]	0.123	.031	.096

Predictor	OR [95% CI]	*SE* (coefficient scale)	Raw *p*	BH-adjusted *p*
Perceived food-safety risk	1.151 [0.805, 1.645]	0.182	.441	.672
Negative environmental impact	0.859 [0.621, 1.189]	0.166	.360	.607
Cooking confidence	1.756 [1.282, 2.406]	0.161	< .001	.003
Curiosity	2.082 [1.578, 2.748]	0.141	< .001	< .001
Worry	0.640 [0.463, 0.886]	0.166	.007	.036
Aversion	0.925 [0.679, 1.261]	0.158	.623	.741
Subjective knowledge	1.498 [1.092, 2.056]	0.161	.012	.051
Factual recognition	1.064 [0.830, 1.365]	0.127	.623	.741
Age	1.185 [0.881, 1.594]	0.151	.261	.561
Female (reference: male)	1.112 [0.664, 1.863]	0.263	.686	.749
Student	0.792 [0.413, 1.521]	0.333	.484	.672
Meat reduction (reference: not reducing)	1.924 [1.067, 3.469]	0.301	.030	.096
Other diet (reference: meat without reduction)	0.586 [0.184, 1.859]	0.589	.364	.607
Bachelor’s (reference: below bachelor’s)	1.140 [0.601, 2.162]	0.327	.689	.749
Master’s or above (reference: below bachelor’s)	0.757 [0.317, 1.808]	0.444	.531	.699
Urban (reference: town/rural)	0.598 [0.281, 1.270]	0.385	.181	.452
Income 3,000–5,999 (reference: <3,000)	0.899 [0.325, 2.486]	0.519	.837	.837
Income 6,000–9,999 (reference: <3,000)	0.697 [0.264, 1.840]	0.495	.466	.672
Income 10,000–14,999 (reference: <3,000)	0.475 [0.175, 1.289]	0.509	.144	.400
Income 15,000–19,999 (reference: <3,000)	1.161 [0.338, 3.993]	0.630	.812	.837
Income ≥20,000 (reference: <3,000)	0.547 [0.175, 1.705]	0.580	.298	.573
Income unknown/withheld (reference: <3,000)	0.318 [0.042, 2.429]	1.037	.270	.561

Unable-to-judge-inclusive logistic — Purchase consideration (n = 1,181)

**Predictor**	**OR [95% CI]**	***SE* (coefficient scale)**	**Raw *p***	**BH-adjusted *p***
Expected taste fit	2.140 [1.618, 2.831]	0.143	< .001	< .001
Expected nutritional value	1.757 [1.340, 2.303]	0.138	< .001	< .001
Perceived naturalness	1.323 [1.066, 1.641]	0.110	.011	.055
Perceived food-safety risk	1.056 [0.776, 1.437]	0.157	.728	.883
Negative environmental impact	0.933 [0.706, 1.233]	0.142	.626	.853
Cooking confidence	1.791 [1.354, 2.370]	0.143	< .001	< .001
Curiosity	2.248 [1.749, 2.891]	0.128	< .001	< .001
Worry	0.616 [0.467, 0.814]	0.142	< .001	.004
Aversion	0.916 [0.701, 1.199]	0.137	.524	.828
Subjective knowledge	1.388 [1.063, 1.814]	0.136	.016	.069
Factual recognition	1.059 [0.846, 1.326]	0.115	.616	.853
Age	1.163 [0.918, 1.473]	0.121	.210	.485
Female (reference: male)	1.129 [0.724, 1.761]	0.227	.592	.853
Student	0.773 [0.439, 1.360]	0.288	.372	.656
Meat reduction (reference: not reducing)	1.727 [1.067, 2.794]	0.246	.026	.098
Other diet (reference: meat without reduction)	0.624 [0.225, 1.732]	0.521	.365	.656
Bachelor’s (reference: below bachelor’s)	1.021 [0.585, 1.782]	0.284	.941	.974
Master’s or above (reference: below bachelor’s)	0.838 [0.374, 1.875]	0.411	.667	.870
Urban (reference: town/rural)	0.593 [0.311, 1.128]	0.328	.111	.303
Income 3,000–5,999 (reference: <3,000)	1.080 [0.456, 2.562]	0.440	.861	.956
Income 6,000–9,999 (reference: <3,000)	0.864 [0.368, 2.025]	0.435	.736	.883
Income 10,000–14,999 (reference: <3,000)	0.592 [0.248, 1.414]	0.444	.238	.510

Predictor	OR [95% CI]	*SE* (coefficient scale)	Raw *p*	BH-adjusted *p*
Income 15,000–19,999 (reference: <3,000)	1.010 [0.355, 2.873]	0.534	.986	.986
Income ≥20,000 (reference: <3,000)	0.583 [0.219, 1.547]	0.498	.278	.556
Income unknown/withheld (reference: <3,000)	0.792 [0.126, 4.988]	0.939	.804	.928
Expected taste fit: unable to judge	0.606 [0.344, 1.067]	0.289	.083	.248
Expected nutritional value: unable to judge	0.415 [0.125, 1.371]	0.610	.149	.373
Perceived naturalness: unable to judge	0.931 [0.162, 5.358]	0.893	.936	.974
Perceived food-safety risk: unable to judge	0.697 [0.265, 1.837]	0.494	.466	.776
Negative environmental impact: unable to judge	0.395 [0.171, 0.913]	0.427	.030	.099

Purchase-minus-trying difference-score OLS — Purchase minus trying (n = 990)

Predictor	*B* [95% CI]	*SE* (coefficient scale)	Raw *p*	BH-adjusted *p*
Expected taste fit	0.069 [0.011, 0.127]	0.030	.020	.135
Expected nutritional value	0.030 [−0.022, 0.082]	0.027	.256	.427
Perceived naturalness	0.030 [−0.020, 0.081]	0.026	.234	.427
Perceived food-safety risk	−0.003 [−0.064, 0.059]	0.031	.932	.932
Negative environmental impact	0.041 [−0.016, 0.098]	0.029	.161	.367
Cooking confidence	0.018 [−0.040, 0.076]	0.030	.551	.689
Curiosity	−0.046 [−0.100, 0.008]	0.028	.097	.304
Worry	−0.069 [−0.130, −0.009]	0.031	.025	.135
Aversion	0.069 [0.009, 0.129]	0.030	.024	.135
Subjective knowledge	−0.028 [−0.080, 0.024]	0.027	.289	.452
Factual recognition	0.025 [−0.025, 0.075]	0.026	.333	.470

**Predictor**	***B* [95% CI]**	***SE* (coefficient scale)**	**Raw *p***	**BH-adjusted *p***
Age	0.005 [−0.048, 0.057]	0.027	.865	.901
Female (reference: male)	0.010 [−0.083, 0.103]	0.048	.837	.901
Student	−0.147 [−0.283, −0.010]	0.069	.035	.145
Meat reduction (reference: not reducing)	0.075 [−0.023, 0.173]	0.050	.131	.352
Other diet (reference: meat without reduction)	0.048 [−0.143, 0.240]	0.098	.622	.709
Bachelor’s (reference: below bachelor’s)	0.065 [−0.046, 0.177]	0.057	.251	.427
Master’s or above (reference: below bachelor’s)	−0.073 [−0.224, 0.078]	0.077	.344	.470
Urban (reference: town/rural)	−0.066 [−0.205, 0.074]	0.071	.357	.470
Income 3,000–5,999 (reference: <3,000)	−0.111 [−0.296, 0.074]	0.094	.240	.427
Income 6,000–9,999 (reference: <3,000)	−0.219 [−0.413, −0.025]	0.099	.027	.135
Income 10,000–14,999 (reference: <3,000)	−0.219 [−0.412, −0.026]	0.099	.026	.135
Income 15,000–19,999 (reference: <3,000)	−0.159 [−0.370, 0.052]	0.108	.141	.352
Income ≥20,000 (reference: <3,000)	−0.199 [−0.413, 0.016]	0.110	.070	.250
Income unknown/withheld (reference: <3,000)	−0.129 [−0.644, 0.386]	0.263	.624	.709

S3.5. Complete multiple-response distributions

Q7

Response option	*n*	Denominator	%
Short videos or social media	665	721	92.2
News reports or popular-science information	457	721	63.4
E-commerce, packaging or advertising	302	721	41.9
Physical shops or restaurants	134	721	18.6
School courses, academic articles or work	120	721	16.6
Family or friends	118	721	16.4
Other	11	721	1.5
Cannot remember	3	721	0.4

Q25

Response option	*n*	Denominator	%
Fit with my taste preferences	690	1,197	57.6
Food safety	919	1,197	76.8
Suitability for my own physical condition	314	1,197	26.2
Nutrition	537	1,197	44.9
Ingredients and production process	252	1,197	21.1
Price	331	1,197	27.7
Convenience of purchasing	79	1,197	6.6
How to cook it or combine it with other foods	219	1,197	18.3
Acceptance by people I eat with	57	1,197	4.8
Value compared with existing foods	113	1,197	9.4
Other	2	1,197	0.2
Nothing in particular matters to me at present	1	1,197	0.1
I cannot say at present	0	1,197	0.0

Q26

Response option	*n*	Denominator	%
What the ingredients are	402	1,197	33.6
Production and processing methods	328	1,197	27.4
Composition and nutrition	824	1,197	68.8
Safety evaluation and suitable consumer groups	749	1,197	62.6
Taste and texture	603	1,197	50.4
Cooking, combining with other foods and storage	258	1,197	21.6
Price and purchasing channels	233	1,197	19.5
Evidence of environmental impact	87	1,197	7.3
Other	0	1,197	0.0
I do not currently want further information	2	1,197	0.2

S3.6. Paired-comparison computation

Paired bootstrap intervals used 5,000 respondent-level resamples with NumPy default_rng seed 20260919. Impression and intention comparisons were executed sequentially with paired responses preserved. Exact two-sided sign and McNemar tests used binomial tail probabilities at probability 0.5. Results are reported in manuscript Sections 3.3–3.4.

Additional paired summaries: among respondents rating both intention items, mean trying and purchase-consideration scores were 4.15 and 3.92 (n = 1,180). In the full sample, 147 respondents expressed positive trying without positive purchase consideration, compared with 19 showing the reverse pattern. The Wilson 95% confidence intervals for the full-sample positive proportions were [84.7%, 88.5%] for trying and [73.5%, 78.4%] for purchase consideration. The full impression transition matrix is reported in manuscript Table 1.

S3.7. Exploratory conditional proportions

Positive responses are scores 4–5. The table below reports conditional proportions connecting prior subjective knowledge (Q5), positive trying (Q13), positive post-description overall impressions (Q12) and explicit inability to judge taste (Q16). Knowledge groups use the verbal anchors of Q5 (1–2, 3, and 4–5). Panel A describes the composition of groups giving positive responses; Panel B describes responses within knowledge groups, making their differing base sizes explicit; Panel C reports recognition of both production facts within the willing-to-try and subjective-knowledge groups. Intervals are pointwise Wilson intervals.

Table S3.7. Exploratory conditional descriptions of acceptance, knowledge and inability to judge taste.

Panel	Conditioning group	Response described	*n*/*N*	%	95% CI
A	Positive willingness to try (Q13 = 4–5)	Little or no subjective knowledge (Q5 = 1–2)	586/1,038	56.5	[53.4, 59.4]
A	Positive post-description overall impression (Q12 = 4–5)	Unable to judge expected taste fit (Q16)	84/979	8.6	[7.0, 10.5]
B	Little or no knowledge (Q5 = 1–2)	Positive willingness to try (Q13 = 4–5)	586/726	80.7	[77.7, 83.4]
B	Little or no knowledge (Q5 = 1–2)	Unable to judge expected taste fit (Q16)	120/726	16.5	[14.0, 19.4]
B	Some knowledge (Q5 = 3)	Positive willingness to try (Q13 = 4–5)	315/332	94.9	[92.0, 96.8]
B	Some knowledge (Q5 = 3)	Unable to judge expected taste fit (Q16)	25/332	7.5	[5.2, 10.9]
B	Quite or very knowledgeable (Q5 = 4–5)	Positive willingness to try (Q13 = 4–5)	137/139	98.6	[94.9, 99.6]
B	Quite or very knowledgeable (Q5 = 4–5)	Unable to judge expected taste fit (Q16)	2/139	1.4	[0.4, 5.1]
C	Positive willingness to try (Q13 = 4–5)	Both production facts recognised (Q9–Q10)	701/1,038	67.5	[64.6, 70.3]
C	Little or no knowledge (Q5 = 1–2)	Both production facts recognised (Q9–Q10)	374/726	51.5	[47.9, 55.1]
C	Some knowledge (Q5 = 3)	Both production facts recognised (Q9–Q10)	268/332	80.7	[76.1, 84.6]
C	Quite or very knowledgeable (Q5 = 4–5)	Both production facts recognised (Q9–Q10)	123/139	88.5	[82.1, 92.8]

Note. Groups overlap. Each row uses its stated denominator; intervals are expressed in percentage units.

S3.8. Subjective knowledge and two-item factual recognition

The table retains all three factual-recognition scores defined in S2.4. The subjective-knowledge groups partition the sample; the willing-to-try group overlaps them. Frequencies within each group sum to its stated denominator.

Table S3.8. Factual recognition by subjective knowledge and willingness to try.

Group	N	Neither fact correct, n (%)	One fact correct, n (%)	Both facts correct, n (%)
All eligible	1,197	162 (13.5)	270 (22.6)	765 (63.9)
Positive willingness to try	1,038	113 (10.9)	224 (21.6)	701 (67.5)
Little or no subjective knowledge	726	145 (20.0)	207 (28.5)	374 (51.5)
Some subjective knowledge	332	13 (3.9)	51 (15.4)	268 (80.7)
Quite or very knowledgeable	139	4 (2.9)	12 (8.6)	123 (88.5)

S3.9. Extended model diagnostics

OLS diagnostics used leverage and Cook's distance from the fitted design matrix. The conventional 4/N Cook's-distance flag was used to describe influence, with all observations retained. Exact leave-one-out coefficient changes were calculated using the OLS deletion identity and expressed relative to the full-model HC3 standard error.

OLS model	Outcome	N	Maximum leverage	Maximum Cook's D	Count above 4/N	Maximum deletion change / HC3 SE
Complete-case OLS	Trying	994	0.138	0.038	49	0.555
Unable-to-judge-inclusive OLS	Trying	1,194	0.137	0.027	58	0.522
Complete-case OLS	Purchase consideration	990	0.150	0.027	49	0.487
Unable-to-judge-inclusive OLS	Purchase consideration	1,181	0.151	0.020	64	0.513
Difference-score OLS	Purchase minus trying	990	0.150	0.027	32	0.621

Logistic diagnostics recorded the number of iterations, maximum absolute score-gradient element, minimum eigenvalue of the observed information matrix, coefficient magnitude and fitted probability range. All four fits converged with finite coefficients, maximum absolute score gradients below 0.000001 and positive minimum information eigenvalues.

**Logistic model**	**Outcome**	**Positive / other scored responses**	**Iterations**	**Minimum informatio n eigenvalue**	**Maximum absolute coefficient**
Complete-case logistic	Trying	900 / 94	8	0.463	3.951
Unable-to-judge-inclusive logistic	Trying	1,038 / 156	8	0.609	3.332
Complete-case logistic	Purchase consideration	818 / 172	7	0.708	3.304
Unable-to-judge-inclusive logistic	Purchase consideration	910 / 271	7	0.959	2.874

S3.10. Pairwise correlations

Spearman correlations use available scorable pairs, excluding unable-to-judge/decide responses only from the corresponding pair. The table reports ρ and pairwise *N*.

Table S3.10. Pairwise Spearman correlations with acceptance intentions.

**Variable**	**Trying: ρ (*n*)**	**Purchase consideration: ρ (*n*)**
Expected taste fit	0.425 (1,049)	0.526 (1,044)
Expected nutritional value	0.389 (1,163)	0.426 (1,151)
Perceived naturalness	0.294 (1,174)	0.349 (1,164)
Perceived food-safety risk	−0.331 (1,147)	−0.331 (1,137)
Negative environmental impact	−0.298 (1,127)	−0.244 (1,120)
Cooking confidence	0.452 (1,194)	0.498 (1,181)
Curiosity	0.444 (1,194)	0.422 (1,181)
Worry	−0.368 (1,194)	−0.373 (1,181)
Aversion	−0.366 (1,194)	−0.302 (1,181)
Subjective knowledge	0.323 (1,194)	0.324 (1,181)
Factual recognition	0.186 (1,194)	0.232 (1,181)

S3.11. Preparation confidence, safety priorities and taste information

Moderate-or-lower cooking confidence is Q21 = 1–3 and low perceived risk is Q19 = 1–2. Safety priority and taste-information requests are selections in Q25 and Q26. Intervals are pointwise Wilson intervals. Groups overlap.

Table S3.11a. Conditional preparation, safety and information profiles.

Conditioning group	Response described	*n*/*N*	%	95% CI
Positive trying	Moderate or lower cooking confidence	532/1,038	51.3	[48.2, 54.3]
Positive purchase consideration	Moderate or lower cooking confidence	421/910	46.3	[43.0, 49.5]
Safety selected as decision priority	Low perceived safety risk	619/919	67.4	[64.3, 70.3]
Unable to judge expected taste fit	Taste/texture information selected	70/147	47.6	[39.7, 55.7]
Scorable expected taste fit	Taste/texture information selected	533/1,050	50.8	[47.7, 53.8]

Among respondents with scorable purchase consideration, attribute-complete and attribute-incomplete groups had positive responses of 818/990 (82.6%) and 92/191 (48.2%), respectively; a further 16 respondents could not decide on purchase consideration.

Table S3.11b. Cooking confidence among all respondents and those expressing positive intentions.

Group	*N*	Low (1–2), *n* (%)	Moderate (3), *n* (%)	High (4–5), *n* (%)
All eligible	1,197	343 (28.7)	335 (28.0)	519 (43.4)
Positive willingness to try	1,038	230 (22.2)	302 (29.1)	506 (48.7)
Positive purchase consideration	910	150 (16.5)	271 (29.8)	489 (53.7)

Note. Percentages use the stated group denominator; groups overlap.

References

2. Hoek, A.C.; Luning, P.A.; Weijzen, P.; Engels, W.; Kok, F.J.; de Graaf, C. Replacement of meat by meat substitutes. A survey on person- and product-related factors in consumer acceptance. *Appetite* **2011**, *56*, 662–673. https://doi.org/10.1016/j.appet.2011.02.001.

3. Michel, F.; Hartmann, C.; Siegrist, M. Consumers' associations, perceptions and acceptance of meat and plant-based meat alternatives. *Food Qual. Prefer.* **2021**, *87*, 104063. https://doi.org/10.1016/j.foodqual.2020.104063.

6. Chezan, D.; Flannery, O.; Patel, A. Factors affecting consumer attitudes to fungi-based protein: A pilot study. *Appetite* **2022**, *175*, 106043. https://doi.org/10.1016/j.appet.2022.106043.

7. Hellwig, C.; Taherzadeh, M.J. Fungi-based food in the public eye: Terminology, cultivation timelines, sustainability, and nutrition across three EU countries. *PLoS ONE* **2026**, *21*, e0345657. https://doi.org/10.1371/journal.pone.0345657.

8. National Health Commission of the People's Republic of China. *Announcement on 14* N*ew* F*ood* I*ngredients,* F*ood* A*dditives and* F*ood*-R*elated* P*roducts,* I*ncluding* M*ycoprotein from Fusarium venenatum* (Announcement No. 7 of 2025). 2025. Available online: https://zwfw.nhc.gov.cn/kzx/zcfg/sptjjxpzsp_236/202512/t20251210_3204.html (accessed on 30 September 2026).

18. Lwin, M.O.; Malik, S.; Ho, J.Y.; Schulz, P.J. Feeding the future: A comparison of drivers and barriers towards consumers' acceptance of plant-based protein, mycoprotein, cultured meat, and insect-based protein in Singapore. *Food Qual. Prefer.* **2026**, *137*, 105761. https://doi.org/10.1016/j.foodqual.2025.105761.

20. Raju, P.S.; Lonial, S.C.; Mangold, W.G. Differential effects of subjective knowledge, objective knowledge, and usage experience on decision making: An exploratory investigation. *J. Consum. Psychol.* **1995**, *4*, 153–180. https://doi.org/10.1207/s15327663jcp0402_04.

22. Tan, H.S.G.; Fischer, A.R.H.; Tinchan, P.; Stieger, M.; Steenbekkers, L.P.A.; van Trijp, H.C.M. Insects as food: Exploring cultural exposure and individual experience as determinants of acceptance. *Food Qual. Prefer.* **2015**, *42*, 78–89. https://doi.org/10.1016/j.foodqual.2015.01.013.

24. Elzerman, J.E.; Hoek, A.C.; van Boekel, M.A.J.S.; Luning, P.A. Consumer acceptance and appropriateness of meat substitutes in a meal context. *Food Qual. Prefer.* **2011**, *22*, 233–240. https://doi.org/10.1016/j.foodqual.2010.10.006.

25. Schösler, H.; de Boer, J.; Boersema, J.J. Can we cut out the meat of the dish? Constructing consumer-oriented pathways towards meat substitution. *Appetite* **2012**, *58*, 39–47. https://doi.org/10.1016/j.appet.2011.09.009.

26. Wilks, M.; Phillips, C.J.C. Attitudes to in vitro meat: A survey of potential consumers in the United States. *PLoS ONE* **2017**, *12*, e0171904. https://doi.org/10.1371/journal.pone.0171904.
